# Iron-based magnetic nanoplatforms for immune microenvironment remodeling and cancer immunotherapy: progress and prospects

**DOI:** 10.1186/s12951-026-04629-2

**Published:** 2026-06-04

**Authors:** Le Chang, Yiqi Yan, Cuixiang Xu, Jianhua Wang

**Affiliations:** 1 Shaanxi Provincial Key Laboratory of Infection and Immune Diseases, Shaanxi Provincial People’s Hospital, Xi’an, 710068 China; 2 Shaanxi Engineering Research Center of Cell Immunology, Shaanxi Provincial People’s Hospital, Xi’an, 710068 China; 3 Department of Graduate School, Yan’an University, Yan’an, 716000 China; 4 Second Department of General Surgery, Shaanxi Provincial People’s Hospital, Xi’an, 710068 China; 5 Bioinspired Engineering and Biomechanics Center (BEBC), Xi’an Jiaotong University, Xi’an, 710049 China

**Keywords:** Iron-based magnetic nanoparticles, Tumor immune microenvironment, Ferroptosis, Immunosuppression, Tumor-associated macrophages, Effector T cells

## Abstract

Cancer continues to be one of the leading causes of death worldwide. Although immunotherapy has transformed oncologic treatment by mobilizing the host immune system, the immunosuppressive tumor immune microenvironment (TIME) still constitutes a major barrier to durable clinical responses. Recent studies indicate that iron-based magnetic nanoparticles (Fe-MNPs) offer capabilities that extend beyond those of conventional drug delivery systems, acting instead as active regulators of the TIME. In this review, we outline current progress in Fe-MNP-mediated cancer immunotherapy and propose a conceptual framework to clarify their immunomodulatory effects. Specifically, Fe-MNPs can generate multiple physicochemical stimuli under magnetic field control, including magnetic hyperthermia, magnetically induced mechanical perturbation, and Fenton reaction-mediated oxidative stress. These upstream events converge to induce immunogenic stress, including ferroptosis-associated injury, which in turn promotes hypoxia alleviation, tumor-associated macrophage (TAM) reprogramming, dendritic cell activation, and enhanced effector T-cell responses. Together, these findings support a framework in which Fe-MNP-mediated immunomodulation is understood as an integrated cascade rather than a collection of isolated effects. However, challenges remain regarding biosafety, long-term iron metabolism, delivery efficiency, and large-scale manufacturing. Overall, this review provides a systematic overview of Fe-MNP-mediated immunomodulation and discusses the translational prospects of these materials as multifunctional platforms for cancer immunotherapy.

**Graphical Abstract:**

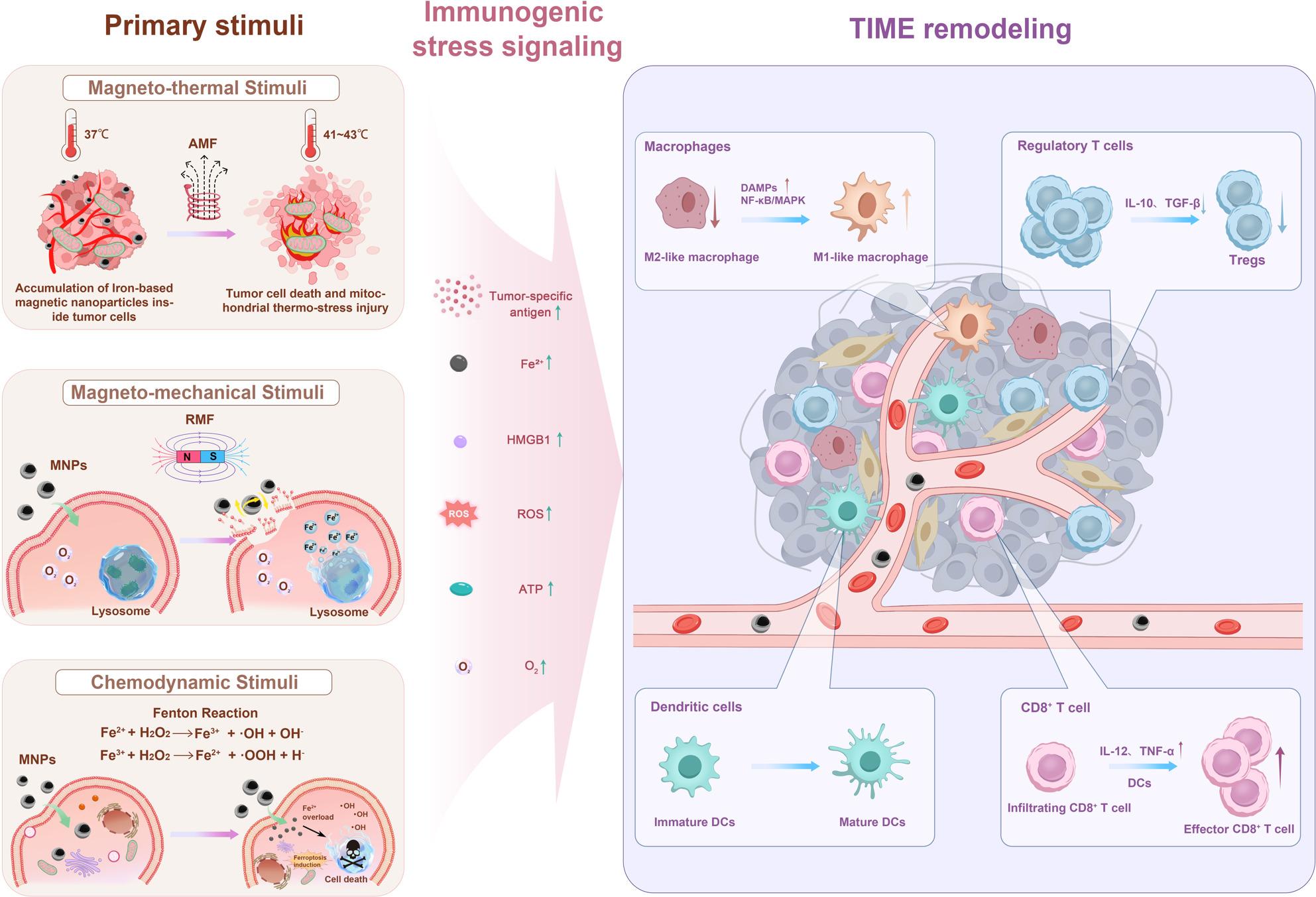

## Highlights

1. Summarizes the physicochemical properties and immune modulation of Fe-MNPs
2. Describes the mechanisms of Fe-MNPs in remodeling the tumor immune microenvironment
3. Explores the potential of Fe-MNPs in combination therapies
4. Proposes a forward-looking framework for future Fe-MNP-based cancer immunotherapy

## Introduction

The tumor microenvironment (TME) is a complex and dynamic ecosystem that supports the survival, proliferation, and evolution of cancer cells [1]. Within this milieu, the immune compartment collectively constitutes the tumor immune microenvironment (TIME), which progressively evolves into an immunosuppressive niche during tumor development. The TIME comprises a diverse array of immune and stromal components, including tumor-associated macrophages (TAMs), dendritic cells (DCs), effector T cells, myeloid-derived suppressor cells (MDSCs), cancer-associated fibroblasts (CAFs), and various resident stromal and parenchymal cell types [2–4]. These cellular components can be broadly classified into anti-tumor and pro-tumor populations [5]. Anti-tumor immune cells, including M1-like TAMs, CD4⁺ and CD8⁺ T lymphocytes, and DCs, enhance antitumor immunity by promoting immune activation and tumor cell elimination [6]. In contrast, immunosuppressive populations such as M2-like TAMs, MDSCs, and regulatory T cells (Tregs) contribute to tumor immune evasion by dampening immune responses. Importantly, these immune cells continuously sense biochemical and biophysical cues in the tumor milieu and initiate distinct functional programs accordingly [7, 8]. Tumor-derived metabolites and altered biochemical signals can, in turn, reciprocally regulate immune cell differentiation, polarization, and activity. Thus, the dynamic interplay among metabolic states, physicochemical cues, and immune cell subsets collectively determines the outcome of antitumor immunity.

In cancer immunotherapy, the complexity and heterogeneity of the TIME remain major obstacles to durable therapeutic efficacy [9]. Although multiple immunotherapeutic strategies have been developed—including immune checkpoint inhibitors (anti-PD-1 and anti-CTLA-4 antibodies), adoptive cell-based therapies, natural killer (NK) cell therapy, tumor vaccines, and molecularly targeted immunomodulation—the immunosuppressive nature of the TIME continues to severely constrain their effectiveness [10]. For instance, programmed death 1(PD-1)/programmed death-ligand 1(PD-L1) blockade exhibits limited clinical benefit in certain patients, largely owing to the high infiltration of immunosuppressive MDSCs and M2-polarized macrophages, which markedly impair the cytotoxic activity of effector T cells [11]. Therefore, strategies that can reverse TIME-mediated immune suppression and re-establish an immune-permissive state have become a major focus of current research.

With the rapid advancement of nanotechnology, nano-immunotherapy has emerged as a promising approach to address these challenges. Among various nanomaterials, iron-based magnetic nanoparticles (Fe-MNPs) have attracted considerable attention because of their unique physical, chemical, and biological properties. Unlike passive drug carriers, Fe-MNPs possess intrinsic magnetic responsiveness and iron-mediated redox activity, enabling them to function not only as delivery vehicles and magnetic resonance imaging (MRI) contrast agents but also as active platforms for immunomodulation [12, 13]. Through these intrinsic properties, Fe-MNPs can generate controllable physical and chemical stimuli within the tumor microenvironment, thereby influencing multiple aspects of the TIME. Emerging evidence suggests that these stimuli collectively contribute to immune modulation and enhanced antitumor responses, although the underlying mechanisms remain to be systematically integrated [14]. Importantly, these effects should not be viewed as isolated mechanisms, but rather as interconnected outcomes arising from the coordinated action of magnetic stimulation and iron-dependent chemistry.

In this review, we propose a unified framework to integrate the diverse immunomodulatory effects of Fe-MNPs in cancer therapy. Within this framework, we first summarize the primary physicochemical actions of Fe-MNPs, including thermal, mechanical, and iron-dependent oxidative stress mechanisms. We then discuss how these upstream events trigger immunogenic stress signaling and ultimately contribute to downstream immune remodeling, such as hypoxia modulation, macrophage reprogramming, dendritic cell activation, and T-cell responses. We further highlight emerging opportunities for magnetically controlled and image-guided immunotherapy, as well as integrated theranostic approaches, together with the key translational challenges that must be addressed for clinical application.

## Fe-MNPs as multifunctional immunomodulatory platforms: a unified framework

### Key physicochemical properties of Fe-MNPs

Iron-based magnetic nanoparticles (Fe-MNPs) represent a distinctive class of nanomaterials that integrate the intrinsic properties of iron with the functional advantages conferred by nanoscale dimensions and magnetic responsiveness. Typically composed of magnetite (Fe₃O₄) or maghemite (γ-Fe₂O₃), these materials exhibit a set of defining features that underpin their biomedical utility and constitute the material basis for their subsequent immunomodulatory functions [15].

One of the most fundamental characteristics of Fe-MNPs is their superparamagnetic behavior at room temperature [16]. When the particle size is reduced below a critical threshold, they exhibit negligible remanent magnetization in the absence of an external magnetic field but become strongly magnetized upon field application. This property enables externally controllable magnetic actuation in multiple forms. Under an alternating magnetic field (AMF), Fe-MNPs convert magnetic energy into thermal energy through Néel and Brownian relaxation, giving rise to magnetic hyperthermia [17]. Under a rotating magnetic field (RMF), they can act as nano-actuators, generating magneto-mechanical perturbations that enable controllable actuation at the cellular level [18]. Compared with optical or ultrasonic approaches, magnetic fields provide deeper tissue penetration with minimal attenuation, thereby supporting noninvasive intervention in deep-seated tumors.

In parallel with their magnetic responsiveness, Fe-MNPs possess intrinsic iron-mediated redox activity. Under the mildly acidic conditions characteristic of the tumor microenvironment (TME) or following lysosomal degradation, Fe-MNPs can release Fe²⁺ and Fe³⁺ ions, which participate in Fenton and Fenton-like reactions to catalyze the conversion of endogenous hydrogen peroxide (H₂O₂) into highly reactive hydroxyl radicals (•OH). This process provides the chemical basis for oxidative stress, lipid peroxidation, and ferroptosis-related responses in tumor cells [19]. As iron is an essential trace element, iron-based formulations may benefit from relatively well-defined physiological pathways for metabolism and clearance, which is relevant for their potential biomedical application.

Another defining feature of Fe-MNPs is their engineering versatility. Their surfaces can be readily functionalized with polymers, targeting ligands, or biomimetic coatings (e.g., cell membranes), enabling improved colloidal stability, prolonged circulation, enhanced tumor targeting, and the co-delivery of immunomodulatory agents [20]. At the same time, Fe-MNPs exhibit strong magnetic susceptibility and can serve as T₂-weighted contrast agents for magnetic resonance imaging (MRI), allowing real-time monitoring of nanoparticle distribution and treatment response. This combination of therapeutic and imaging capabilities provides a distinctive theranostic advantage.

Taken together, the significance of Fe-MNPs arises not from any single property in isolation, but from the convergence of magnetic controllability, iron-dependent chemical reactivity, and engineering flexibility. These integrated features establish the material basis for the diverse biological effects discussed in the following sections.

### A unified framework for Fe-MNP-mediated immune remodeling

**Fig. 1:**
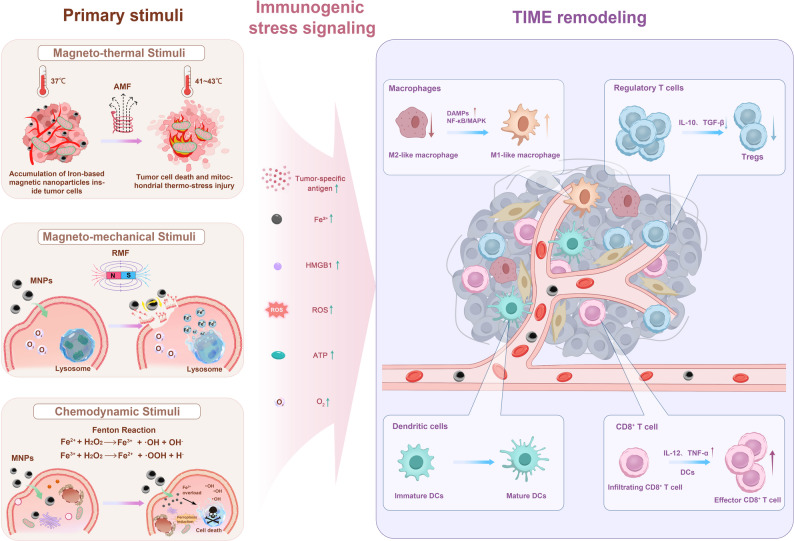
A unified framework for Fe-MNP-mediated remodeling of the tumor immune microenvironment (TIME). Fe-MNPs integrate magnetic responsiveness and iron-dependent chemistry to generate three major categories of primary physicochemical inputs, including magneto-thermal, magneto-mechanical, and chemodynamic stimuli. These upstream inputs converge on localized immunogenic stress signaling, accompanied by reactive oxygen species (ROS) accumulation and the release of danger-associated molecular patterns (DAMPs). The resulting stress-associated signals subsequently contribute to TIME remodeling by promoting macrophage repolarization, dendritic cell maturation, reduced regulatory T-cell-associated immunosuppression, and enhanced CD8⁺ T-cell infiltration and activation

Building on these defining features, a conceptual framework is needed to explain how Fe-MNPs coordinate immune remodeling. Existing studies often discuss magnetic hyperthermia, mechanical disruption, ferroptosis induction, TAM reprogramming, and hypoxia relief as separate functions. However, these processes are biologically connected through the combined effects of magnetic responsiveness, iron-dependent chemistry, and engineering adaptability. In this context, Fe-MNPs can be conceptualized as a “thermal–mechanical–chemical” tripartite converter that coordinates a unified spatiotemporal cascade of tumor immune microenvironment (TIME) remodeling (Fig. 1).

At the initial stage, Fe-MNPs translate externally applied magnetic fields and endogenous iron chemistry into multiple forms of physicochemical stimuli. Under magnetic-field regulation, Fe-MNPs can generate two major forms of physical stimulation: magnetothermal input under alternating magnetic fields and magneto-mechanical perturbation under dynamic magnetic fields such as RMF [21, 22]. In parallel, under the mildly acidic conditions of the TME, released iron ions catalyze Fenton and Fenton-like reactions, generating hydroxyl radicals (•OH) and oxidative stress [23]. Together, these inputs constitute the primary driving forces that initiate tumor cell damage.

These distinct physicochemical inputs subsequently converge on a common cellular outcome, namely immunogenic stress signaling. Whether initiated by thermal stimulation, mechanical perturbation, or oxidative lipid damage, Fe-MNP-mediated injury triggers stress responses associated with lipid peroxidation, membrane damage, ferroptosis-related injury, and damage-associated signaling [24, 25]. Such processes are frequently accompanied by the release or exposure of danger-associated molecular patterns (DAMPs), including ATP, high-mobility group box 1 (HMGB1), and calreticulin (CRT), as well as the accumulation of reactive oxygen species (ROS) [26]. These stress-associated signals provide a mechanistic bridge linking direct tumor cell injury to immune activation.

At the level of the TIME, these stress signals initiate a coordinated cascade that connects innate and adaptive immunity [27]. In catalytically active or oxygen-generating Fe-MNP systems, tumor hypoxia can be alleviated, thereby partially restoring local oxygen availability and creating conditions more favorable for immune responsiveness [28]. Tumor-associated macrophages (TAMs) may shift from an immunosuppressive M2-like phenotype toward a more pro-inflammatory state, while dendritic cells (DCs) undergo maturation with enhanced antigen presentation and cross-presentation capacity [29]. Collectively, these changes create a more permissive immune context that supports the recruitment, infiltration, and activation of cytotoxic T lymphocytes while relieving key immunosuppressive constraints [30].

Importantly, these processes are not strictly sequential but are dynamically interconnected and mutually reinforcing. Hypoxia relief facilitates oxidative and ferroptosis-related responses by improving local oxygen availability, while macrophage reprogramming enhances downstream T-cell recruitment through chemokine secretion [14, 31]. In addition, integrating magnetothermal stimulation with catalytic oxidative injury can potentiate ICD-associated stress signaling and DAMP release beyond what is typically achieved by either modality alone [32]. Taken together, these interactions indicate that Fe-MNP-mediated immune remodeling is best understood as an integrated network rather than a linear sequence of isolated events. Unlike platforms that rely mainly on a single mechanism, Fe-MNPs combine magnetically addressable physical stimulation, iron-dependent chemical stress, and imaging compatibility within one platform. This integrated functionality distinguishes them from non-magnetic iron nanoparticles, manganese-based materials, and generic ROS-generating systems, and helps define the technological distinctiveness of magnetic iron platforms in cancer immunotherapy.

In summary, Fe-MNPs function as multifunctional platforms that couple magnetic stimulation with iron-dependent chemistry to generate coordinated physicochemical inputs and downstream immune responses. This unified framework provides a coherent conceptual basis for understanding how Fe-MNPs mediate TIME remodeling and clarifies the interconnections among their primary physicochemical and immunological effects.

## Primary driving mechanisms of Fe-MNP-mediated immune remodeling

### Magnetic hyperthermia-mediated thermal stimulation

**Fig. 2:**
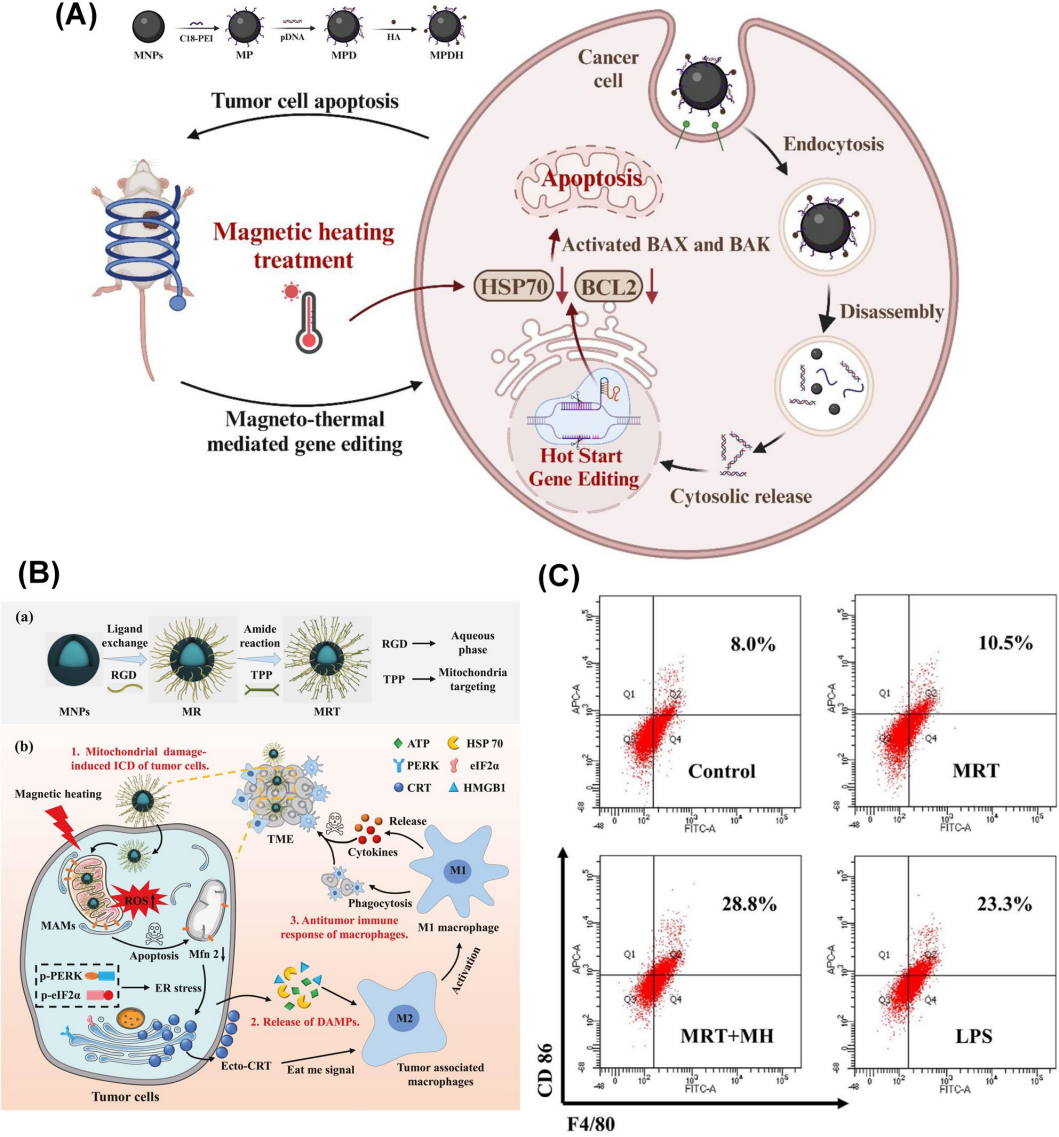
Magnetic hyperthermia-mediated tumor cell injury and immune-associated responses of Fe-MNPs. (**A**) Schematic illustration of Fe-MNP accumulation in tumor tissue and localized hyperthermia under AMF, leading to tumor cell death. Reproduced with permission from Ref. [33]. (**B, C**) Mitochondria-targeted magnetic hyperthermia-induced ICD and flow cytometric analysis of macrophage polarization. Reproduced with permission from Ref. [35]

Within the unified framework described above, magnetic hyperthermia represents one of the primary upstream mechanisms by which Fe-MNPs convert external magnetic fields into biologically active stress signals. Under an alternating magnetic field (AMF), Fe-MNPs dissipate magnetic energy as heat through Néel and Brownian relaxation, resulting in localized temperature elevation within tumor tissues [17]. Owing to their favorable balance of biocompatibility and magnetic responsiveness, Fe-MNPs—particularly iron oxide-based nanomaterials—are widely investigated as agents for magnetic hyperthermia. Following intratumoral or systemic administration, Fe-MNPs can accumulate in tumors through passive retention, magnetic targeting, or ligand-assisted cellular uptake, thereby enabling spatially confined heating under external field stimulation. Such localized hyperthermia typically induces protein denaturation, metabolic disruption, membrane damage, and organelle dysfunction, ultimately leading to tumor cell death. Importantly, beyond its direct cytotoxic effects, this thermally induced stress also provides an upstream basis for immunogenic signaling and subsequent remodeling of the tumor immune microenvironment [17, 33] (Fig. 2A).

Based on the mechanistic basis described above, representative studies further illustrate how Fe-MNP-mediated magnetic hyperthermia progresses from enhanced tumor cell killing to the induction of immune-related responses. Rodríguez-Luccioni et al. reported that magnetic fluid hyperthermia (MFH) induced more pronounced tumor cell death than conventional hot water hyperthermia (HWH). In Caco-2 and MCF-7 cells, MFH reduced cell viability by 40% and 55%, respectively, and increased apoptosis rates by 30% and 60% relative to HWH [34]. More importantly, the biological impact of MFH extends beyond direct tumor cell killing. Jiang et al. developed a mitochondria-targeted magnetic hyperthermia system (MRT) that selectively induced immunogenic cell death (ICD) in tumor cells, accompanied by the release of DAMPs such as ATP and HSP70 (Fig. 2B). Consistent with this immunogenic stress response, flow cytometric analysis further showed that the proportion of F4/80⁺CD86⁺ macrophages increased from 8.0% in the control group to 28.8% in the MRT + MH group (Fig. 2C) [35]. Collectively, these findings indicate that MFH not only exhibits superior tumoricidal efficacy compared with conventional heating approaches, but also initiates early immune-activating events that are relevant to subsequent TIME remodeling.

**Fig. 3:**
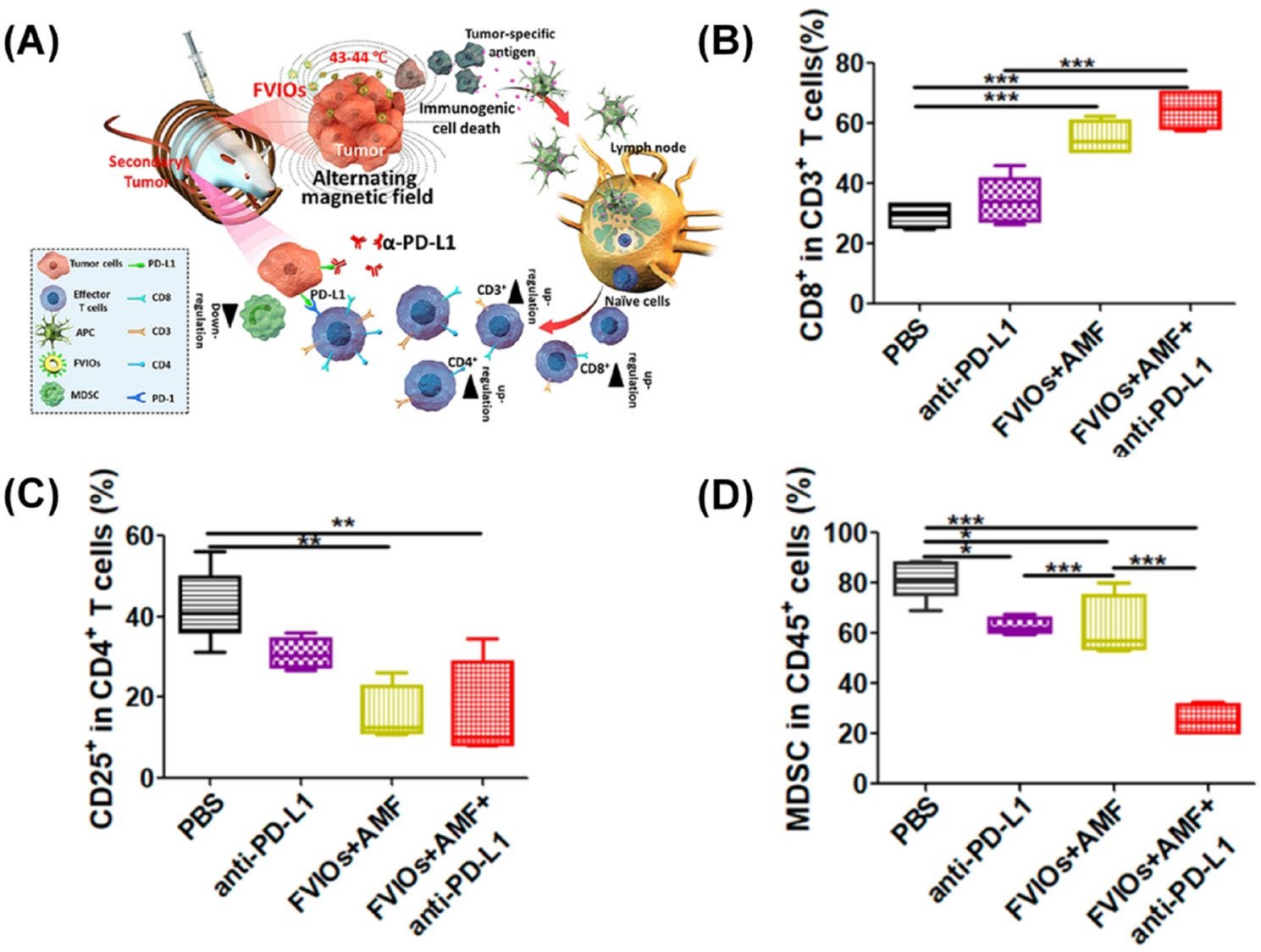
Synergistic antitumor effects of MHT-based combination therapies. (**A**) Schematic illustration of FVIOs mediating MHT to induce ICD and antigen release, which reverses the immunosuppressive tumor microenvironment and potentiates the efficacy of anti-PD-L1 therapy. (**B-D**) Flow cytometry analysis showing the percentage of CD8⁺ T cells, Treg cells, and MDSCs in different treatment groups. Reproduced with permission from Ref. [36]

Beyond its effects on innate immune components, magnetic hyperthermia can also induce early changes relevant to adaptive immunity. Liu et al. synthesized ferrimagnetic vortex iron oxide nanoparticles (FVIOs), which, under alternating magnetic field (AMF) stimulation, induced immunogenic cell death (ICD) and tumor-associated antigen release (Fig. 3A), accompanied by a shift of TAMs toward an M1-like phenotype. Further analysis showed that the proportion of CD8⁺ cytotoxic T lymphocytes increased to 55.4%, whereas Tregs were reduced by 27%, and MDSCs were also downregulated (Fig. 3B–D) [36]. Similar immune-enhancing trends have also been observed in other Fe-MNP platforms, including M1-like macrophage polarization, enhanced T-cell recruitment, increased intratumoral CD8⁺ T-cell responses, and more sustained T-cell activation, suggesting that magnetic hyperthermia-induced immune activation is not restricted to a single nanoparticle architecture or experimental mode [37–39]. Collectively, these findings indicate that Fe-MNP-mediated magnetic hyperthermia not only enhances direct tumor killing, but also initiates immune changes relevant to subsequent TIME remodeling by rebalancing effector and immunosuppressive cell populations.

It should be noted, however, that the immunological consequences of magnetic hyperthermia are not uniformly beneficial and remain highly dependent on the thermal window and spatial control of heating [40, 41]. Mild and well-controlled heating is generally more favorable for improving perfusion, promoting reoxygenation, and enhancing immune activation, whereas excessive or heterogeneous heating may weaken antitumor immune responses. Accordingly, current conclusions should still be interpreted in light of model type, immune context, and thermal dosimetry, as the available evidence remains largely preclinical and varies in strength across different experimental settings.

### Magnetically driven mechanical disruption

Within the unified framework proposed in Sect. "Fe-MNPs as multifunctional immunomodulatory platforms: a unified framework", magnetically driven mechanical disruption represents a second major upstream route through which Fe-MNPs exert biological effects. Unlike magnetic hyperthermia, which relies on alternating magnetic fields to generate heat, this mode of action exploits the ability of Fe-MNPs to translate low-frequency dynamic magnetic fields—particularly rotating magnetic fields (RMF) —into localized mechanical forces at the nanoscale. Under such stimulation, Fe-MNPs may undergo rotation, oscillation, translation, or clustering, thereby generating shear stress and physical perturbations that disrupt cellular membranes, lysosomes, and subcellular structures [22, 42]. In addition to these dynamic mechanical effects, static magnetic fields can also be employed for a distinct but complementary purpose: magnetically guided targeting. By applying an external static magnetic field, Fe-MNPs can be concentrated at tumor sites, thereby enhancing local accumulation and reducing systemic exposure of co-delivered therapeutic agents [43, 44]. Overall, this mode of magnetic actuation provides an important basis for both direct tumor disruption and spatially controlled delivery.

**Fig. 4:**
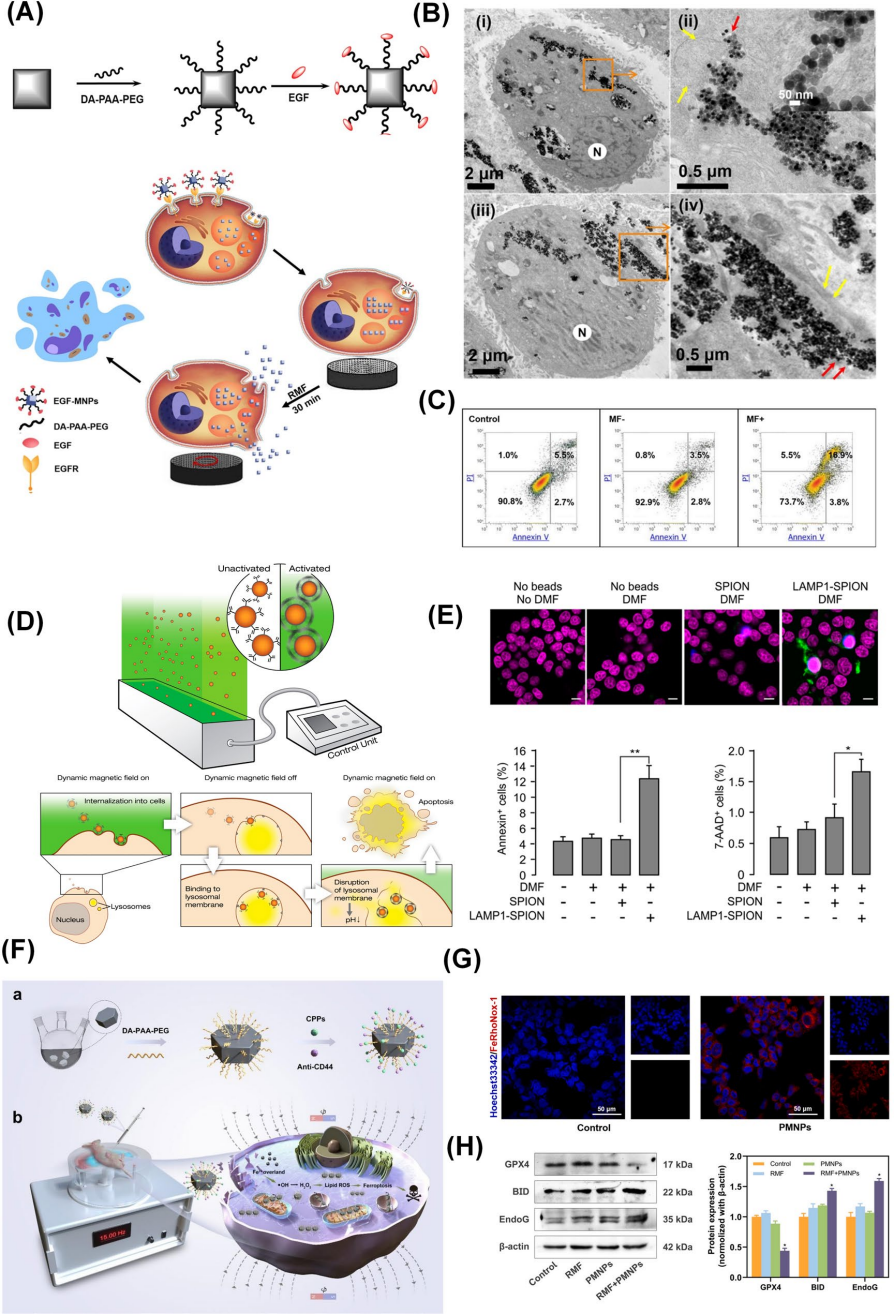
Magnetically actuated Fe-MNPs induce cell death through mechanical disruption. (**A**) Schematic illustrating nanoparticle synthesis, cellular uptake, and RMF-induced membrane damage. (**B**) Transmission electron microscopy images showing disruption of lysosomal and plasma membranes in U87 cells under RMF stimulation. (**C**) Flow cytometric analysis of apoptosis after treatment. Reproduced with permission from Ref. [45]. (**D**) Schematic showing RMF-induced rotation of targeted nanoparticles, leading to LMP and subsequent apoptosis. (**E**) Quantification of increased apoptosis rates in rat insulinoma cells. Reproduced with permission from Ref. [46]. (**F**) Schematic of PMNP-mediated mechanical disruption and ferroptosis-related responses under RMF. (**G**) Immunofluorescence images showing increased intracellular Fe²⁺ levels after treatment. (**H**) Western blot analysis of GPX4, BID, and EndoG expression. Reproduced with permission from Ref. [49]

A primary mode of magnetic actuation is the direct mechanical disruption of cellular membranes and intracellular organelles. Shen et al. developed epidermal growth factor (EGF)-functionalized iron oxide nanoparticles (Fig. 4A), and transmission electron microscopy confirmed that these nanoparticles disrupted both lysosomal and plasma membrane integrity under low-frequency RMF stimulation (Fig. 4B). In glioblastoma U87 cells, this treatment increased early apoptosis by 1.1% and late apoptosis by 11.4% relative to controls (Fig. 4C) [45]. To improve subcellular specificity, Zhang et al. conjugated lysosome-associated membrane protein 1 (LAMP1) antibodies to magnetic nanoparticles, thereby enabling lysosome-targeted mechanical actuation. Under a dynamic magnetic field, these nanoparticles markedly increased lysosomal membrane permeability, leading to lysosomal disruption and intracellular content leakage (Fig. 4D). In rat insulinoma cells, early apoptosis increased from 4.56% ± 0.55% to 12.45% ± 1.6%, whereas late apoptosis rose from 0.73% ± 0.17% to 1.31% ± 0.16% (Fig. 4E) [46].

Importantly, the consequences of such mechanically induced lysosomal damage may extend beyond apoptosis alone. Lysosomal destabilization may release labile Fe²⁺ into the cytosol, thereby promoting Fenton chemistry, lipid peroxidation, and ferroptosis-related injury, while also facilitating the generation of DAMP-associated signals [47, 48]. Consistent with this concept, Wang et al. developed polyhedral magnetic nanoparticles (PMNPs) and showed that, under RMF, these nanoparticles induced both cytotoxicity and ferroptosis in gastric cancer stem-like cells (Fig. 4F). Immunofluorescence demonstrated increased intracellular Fe²⁺ levels after PMNP treatment (Fig. 4G), accompanied by reduced GPX4 expression and 1.2- and 1.4-fold upregulation of BID and EndoG, respectively (Fig. 4H) [49].

Beyond catastrophic membrane failure, magnetic actuation can elicit more subtle, yet equally consequential, biological effects through mechanotransduction. For example, Kim et al. demonstrated that magnetic micro-disks subjected to an AMF generated mechanical torque sufficient to elevate intracellular Ca²⁺ levels without causing immediate membrane rupture, leading to mitochondrial Ca²⁺ overload and subsequent apoptosis [50]. This modulation of Ca²⁺ signaling is highly relevant to immune remodeling, as Ca²⁺ is a critical secondary messenger that activates transcription factors such as the nuclear factor of activated T-cells (NFAT). NFAT activation, in turn, governs the production of pro-inflammatory cytokines essential for robust T-cell responses, offering a precise route to influence the TIME [51]. In addition to these dynamic effects, static magnetic fields enable a distinct but complementary application—magnetically targeted drug delivery—by concentrating drug-loaded Fe-MNPs at tumor sites, thereby enhancing local accumulation while minimizing systemic toxicity [52].

Despite these promising findings, several limitations warrant consideration. The magnitude of mechanical force is highly dependent on nanoparticle concentration, size, spatial distribution, and RMF parameters, making precise and reproducible control challenging. Off-target mechanical effects on surrounding healthy tissues, particularly in complex anatomical sites, have not been systematically evaluated. Moreover, most current evidence is derived from in vitro or subcutaneous tumor models, and the translation of these force-mediated effects to clinically relevant orthotopic or metastatic settings requires further validation. These considerations underscore the need for improved delivery strategies, optimized RMF parameters, and deeper mechanistic understanding of force-induced immunogenic signaling.

Together, these findings indicate that magnetically driven mechanical disruption can act not only through direct structural damage, but also through lysosome-centered stress pathways and mechanosensitive signaling, thereby coupling physical forces to ferroptosis and immunogenic activation.

### Fenton chemistry, ROS generation, and ferroptosis induction

Fenton chemistry constitutes the third major branch of the unified thermal–mechanical–chemical framework introduced in Sect. "Fe-MNPs as multifunctional immunomodulatory platforms: a unified framework". Unlike magnetic hyperthermia or magnetically driven mechanical disruption, which rely on external magnetic fields, this mode of action exploits the intrinsic redox activity of Fe-MNPs to generate oxidative stress within the tumor microenvironment. Under the mildly acidic conditions characteristic of the tumor microenvironment or following lysosomal degradation, Fe-MNPs release Fe²⁺ and Fe³⁺ ions, which catalyze Fenton and Fenton-like reactions and convert endogenous hydrogen peroxide (H₂O₂) into highly reactive hydroxyl radicals (•OH) [53, 54]. The resulting oxidative stress damages proteins, nucleic acids, and membrane lipids, thereby establishing the chemical basis for lipid peroxidation and regulated cell death. In this context, Fe-MNP-mediated chemodynamic activity should be understood as a primary upstream chemical driver of immune remodeling, rather than as an immune outcome per se.

One major consequence of Fenton-driven oxidative stress is ferroptosis, an iron-dependent form of regulated cell death characterized by excessive lipid peroxidation [55]. Mechanistically, ROS attack polyunsaturated phospholipids in cellular membranes, leading to the accumulation of lipid peroxides and progressive membrane dysfunction [56]. This process is normally restrained by antioxidant systems centered on glutathione (GSH) and glutathione peroxidase 4 (GPX4); however, when ROS production exceeds the cellular buffering capacity, or when GPX4 activity is compromised, lipid peroxidation proceeds unchecked and ferroptotic injury ensues [57]. As efficient iron donors and oxidative amplifiers, Fe-MNPs are therefore well suited to trigger ferroptosis in tumor cells and have become prominent agents in chemodynamic therapy (CDT) research.

**Fig. 5:**
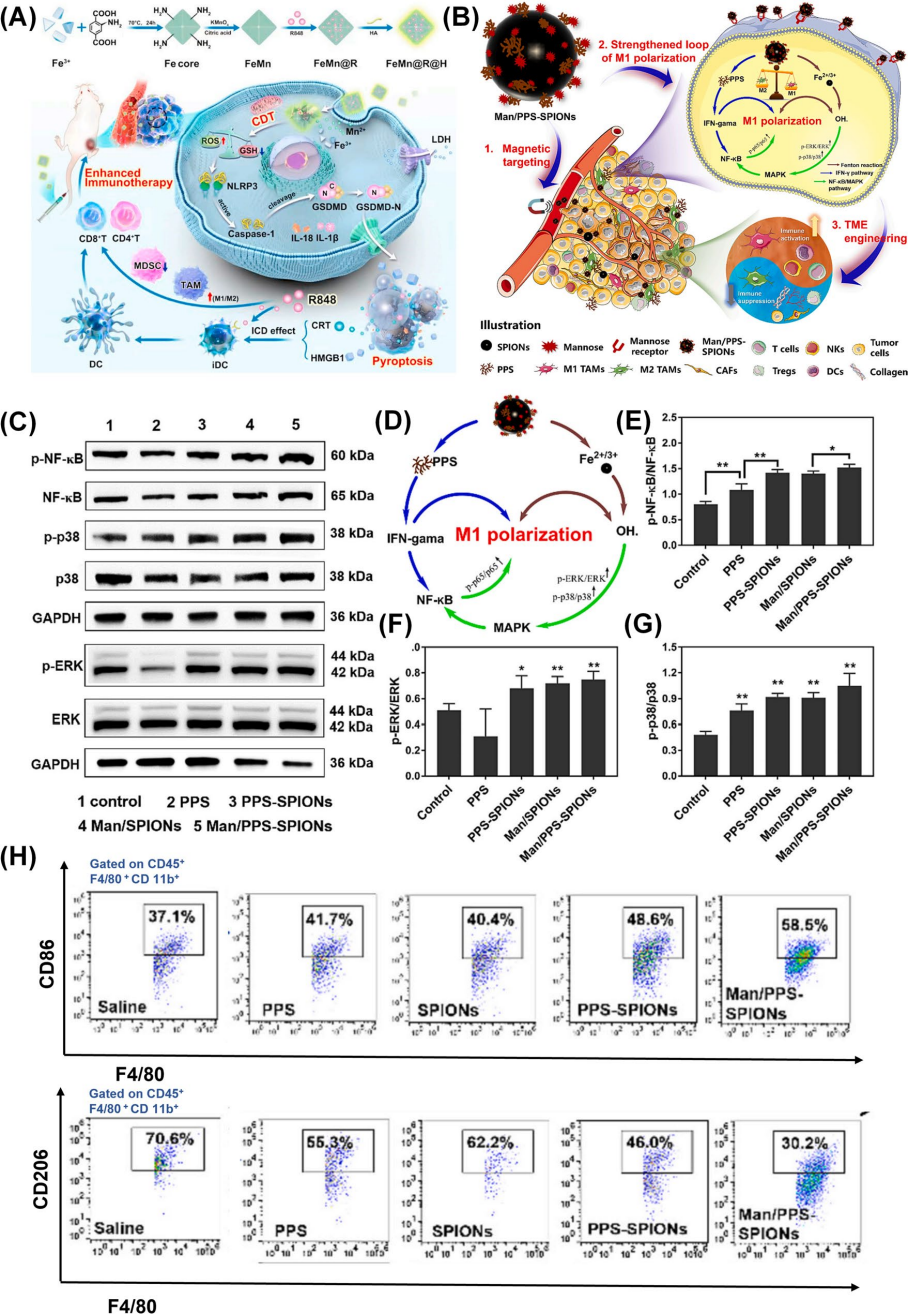
Immune-relevant consequences of Fe-MNP-mediated ROS generation. (**A**) Schematic illustration of FeMn@R@H-induced ROS generation, NLRP3 inflammasome activation, and pyroptosis. Reproduced with permission from Ref. [58]. (**B**) Schematic overview of Man/PPS-SPION-mediated macrophage repolarization. (**C–G**) Western blot analysis and quantification of NF-κB and MAPK pathway-related proteins after treatment. (**H**) Flow cytometric analysis of CD86⁺ M1 macrophages and CD206⁺ M2 macrophages in different treatment groups. Reproduced with permission from Ref. [59]

Representative studies further support the chemical and immune-related relevance of this process. Feng et al. developed a Fe–Mn bimetallic metal-organic framework nanodrug (FeMn@R@H) that generated high levels of ROS through Fenton reactions in 4T1 breast cancer cells, leading to NLRP3 inflammasome activation and Caspase-1-dependent pyroptosis (Fig. 5A) [58]. Likewise, Liu et al. constructed porous superparamagnetic iron oxide nanoparticles coated with mannose and polysaccharides (Man/PPS-SPIONs), which released Fe²⁺ to initiate Fenton reactions and ROS production in a 4T1 breast tumor model (Fig. 5B–G). In this system, ROS-generating CDT was accompanied by a 16.8% increase in M1-type TAMs and a 25.1% decrease in M2-type TAMs compared with PPS monotherapy (Fig. 5H) [59]. These findings suggest that Fenton chemistry and ROS generation act not only as cytotoxic mechanisms, but also as upstream chemical triggers of subsequent TIME remodeling.

Importantly, Fe-MNP-induced ferroptosis should not be interpreted as being exclusively governed by the canonical system Xc⁻/GSH/GPX4 axis [57, 60]. Although GPX4 insufficiency frequently accompanies ferroptotic injury in Fe-MNP systems, additional mechanisms may also contribute, including lysosomal iron mobilization, direct ROS-driven membrane lipid peroxidation, and oxidative amplification in other subcellular compartments [61, 62]. Thus, Fe-MNP-mediated ferroptosis is better understood as a convergent oxidative death program that often involves, but is not necessarily restricted to, the system Xc⁻/GPX4 pathway [63]. At the same time, the immunological consequences of ferroptosis should not be assumed to be uniformly beneficial. While ferroptotic stress in tumor cells may favor immunogenic signaling, excessive or poorly controlled iron-driven oxidative stress may also provoke maladaptive inflammation or impair beneficial immune effectors, indicating that its biological impact is likely to be highly context-dependent [64, 65].

Taken together, Fenton chemistry endows Fe-MNPs with a chemically distinct mode of action that links iron-dependent ROS generation to ferroptosis and early immune-relevant stress. These effects position Fe-MNP-mediated chemodynamic activity as a major upstream chemical driver of TIME remodeling, while also underscoring the need to interpret its immunological consequences with appropriate caution.

## Downstream consequences of Fe-MNP-mediated TIME remodeling

### Reversal of hypoxia and restoration of immune permissiveness

Among the downstream consequences of Fe-MNP-mediated TIME remodeling, reversal of tumor hypoxia occupies a distinct position. Unlike the cell-intrinsic immune changes discussed in the following sections, hypoxia alleviation primarily represents a microenvironmental shift that restores the conditions required for effective immune activation. In this sense, it functions less as a terminal immune outcome than as a prerequisite for subsequent TAM reprogramming, dendritic cell activation, and T-cell infiltration.

Tumor hypoxia is a defining feature of most solid tumors and a major barrier to effective anticancer immunity [66, 67]. It arises from an imbalance between the rapid oxygen consumption of proliferating tumor cells and the insufficient supply provided by structurally abnormal and functionally inefficient tumor vasculature [68, 69]. Beyond compromising the efficacy of radiotherapy and chemotherapy, hypoxia also directly reinforces immune suppression within the tumor microenvironment. In particular, hypoxic stress can promote PD-L1 expression, reduce MHC-I presentation on tumor cells, and support the accumulation or function of immunosuppressive cell populations such as myeloid-derived suppressor cells (MDSCs), thereby limiting cytotoxic T-cell activity and facilitating immune escape [70, 71].

**Fig. 6:**
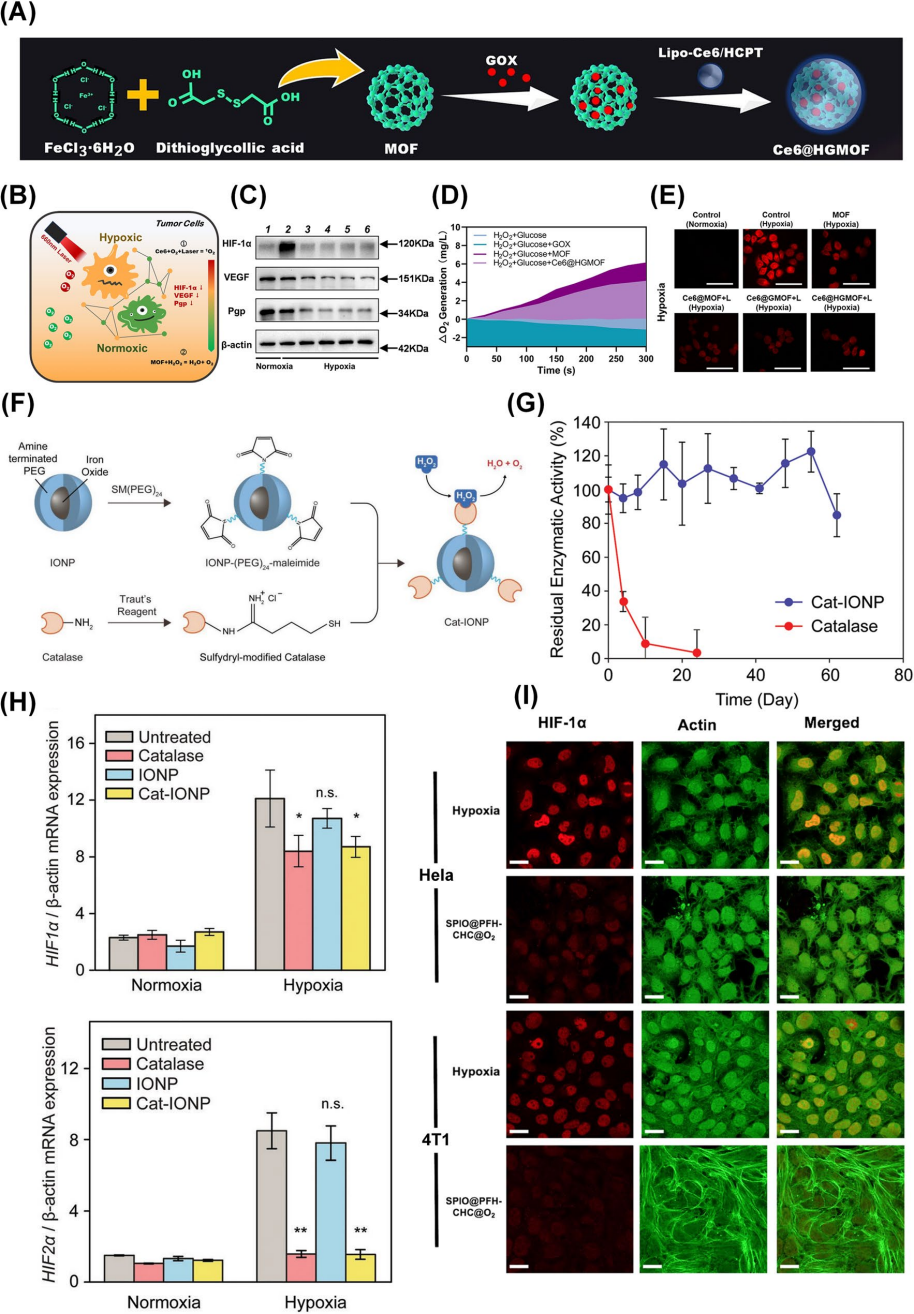
Fe-MNP-based strategies for alleviating tumor hypoxia. (**A**) Schematic illustration of the synthesis of Ce6@HGMOF nanoparticles. (**B**) Schematic of Ce6@HGMOF-mediated relief of tumor hypoxia through catalytic O₂ generation. (**C**) Western blot analysis showing downregulation of HIF-1α and VEGF proteins. (**D**) Dissolved oxygen concentration in different solutions. (**E**) CLSM images of EC109 cells using a hypoxia/ROS detection probe. Reproduced with permission from Ref. [72]. (**F**) Schematic illustration of Cat-IONP synthesis. (**G**) Time-dependent residual enzymatic activity of Cat-IONP and free catalase at pH 7.4. (**H**) qRT-PCR analysis of HIF-1α and HIF-2α mRNA expression under hypoxic conditions. Reproduced with permission from Ref. [73]. (**I**) Immunofluorescence images of HIF-1α expression in 4T1 and HeLa cells after treatment with SPIO@PFH-CHC. Adapted with permission from Ref. [76]

Fe-MNPs provide several complementary strategies to relieve tumor hypoxia and thereby restore a more immune-permissive milieu. One important mechanism is their intrinsic catalase-like activity, which enables the in situ decomposition of endogenous H₂O₂ into O₂. More advanced Fe-based systems have since improved this function. For example, the Fe-based MOF Ce6@HGMOF efficiently generated O₂ under acidic tumor conditions, reduced HIF-1α and VEGF expression, and produced up to 4 mg/L of O₂ within 300 s (Fig. 6A–E) [72]. In addition to their intrinsic catalytic properties, Fe-MNPs can also serve as platforms for delivering exogenous oxygen-generating enzymes or oxygen-carrying components. Yen et al. developed a catalase–iron oxide nanoparticle composite (Cat-IONP) that maintained catalytic activity for up to 60 days and significantly reduced HIF-1α and HIF-2α expression under hypoxic conditions (Fig. 6F–H) [73]. Beyond direct oxygen generation, Fe-MNPs can also be combined with oxygen-carrying molecules such as perfluorocarbons (PFCs) or hemoglobin to further improve oxygen delivery [74, 75]. For example, Chen et al. constructed a hybrid oxygen delivery system (SPIO@PFH-CHC) by encapsulating superparamagnetic iron oxide nanoparticles (SPIO) and perfluorohexane (PFH) within carboxymethyl hexanoyl chitosan (CHC). This system significantly reduced HIF-1α expression in both 4T1 breast cancer and HeLa cervical cancer cells, indicating effective relief of tumor hypoxia (Fig. 6I) [76]. In parallel, mild magnetic hyperthermia may indirectly improve oxygen availability by enhancing local perfusion and vasodilation, thereby increasing vascular oxygen supply.

Collectively, these findings indicate that Fe-MNPs can counteract tumor hypoxia through catalytic, carrier-based, and thermally assisted mechanisms. By relieving oxygen deprivation, these systems may weaken hypoxia-driven immune suppression and restore conditions more favorable for antigen presentation, T-cell activation, and immune infiltration. At the same time, the extent of this benefit remains context-dependent, as most supporting evidence is still preclinical and often relies on surrogate readouts such as HIF-1α downregulation rather than direct measurements of durable immune restoration. Even so, hypoxia reversal represents a critical microenvironmental foundation upon which subsequent immune-cell-specific remodeling can occur.

### Reprogramming of tumor-associated macrophages

Tumor-associated macrophages (TAMs) play a pivotal role in tumor progression and immune evasion, and their abundance is closely associated with poor clinical outcomes in cancer patients [77]. Within the TME, TAMs are often described along a spectrum ranging from proinflammatory, antitumor M1-like states to immunosuppressive, protumor M2-like states. M1-like TAMs secrete proinflammatory cytokines such as TNF-α and interleukin-12 (IL-12), which activate T cells and enhance antitumor immune responses. In contrast, M2-like TAMs release immunosuppressive factors such as transforming growth factor-beta (TGF-β) and VEGF, thereby promoting tumor cell proliferation, angiogenesis, and immune evasion [78, 79]. In most solid tumors, TAMs are enriched in M2-like phenotypes, which are generally associated with reduced responsiveness to immunotherapy [80]. Therefore, reprogramming TAMs toward the M1-like phenotype has emerged as a promising strategy to remodel the TME. Building on the hypoxia reversal discussed in Sect. "Reversal of hypoxia and restoration of immune permissiveness", which restores a more permissive microenvironment, TAM reprogramming represents a critical next step in converting upstream physical and chemical stimuli into innate immune activation.

**Fig. 7:**
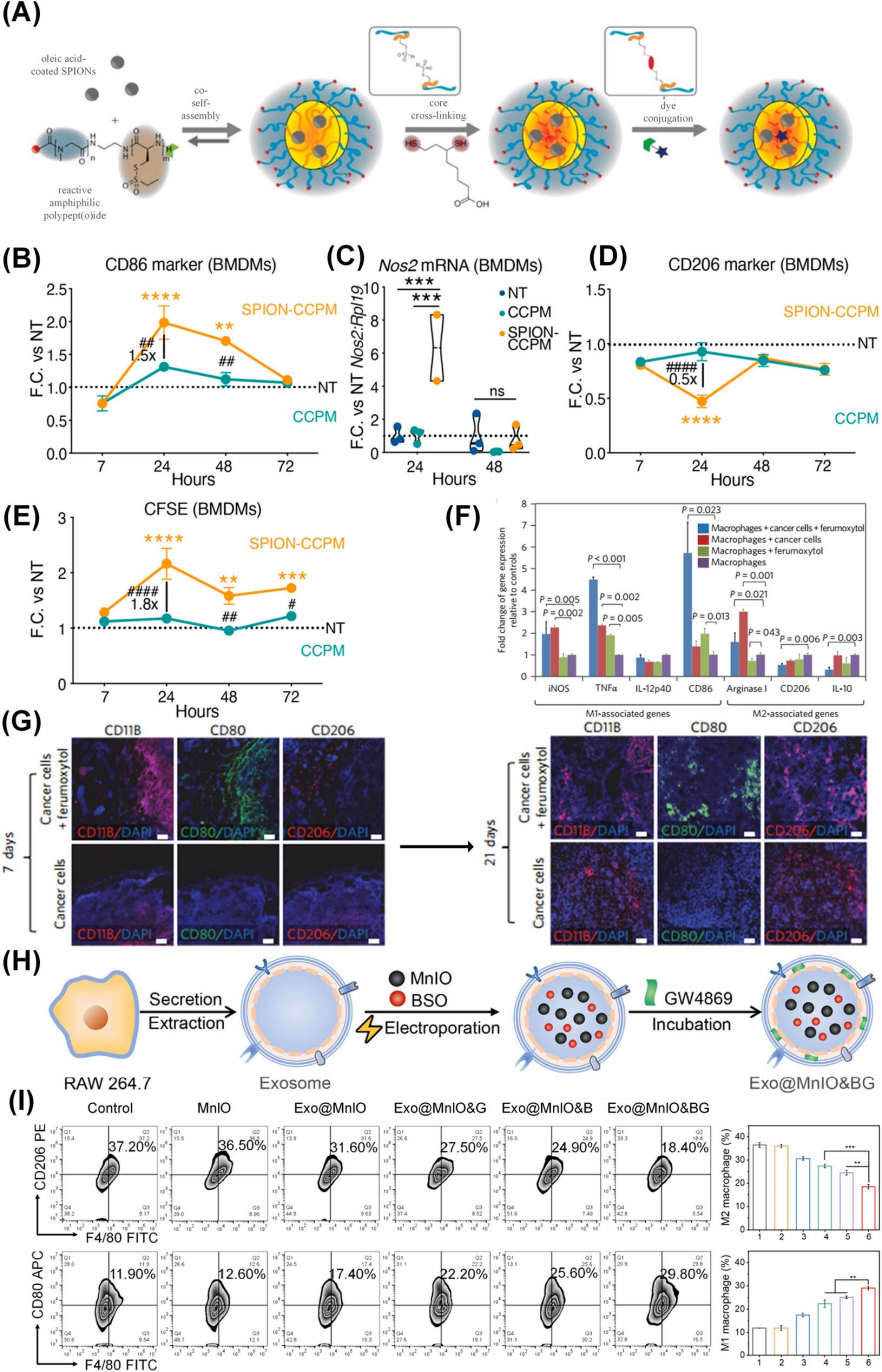
Fe-MNP-based strategies for TAM reprogramming. (**A**) Schematic illustration of SPION-CCPM nanoparticle synthesis. (**B-D**) Analysis of M1-associated markers (CD86, Nos2) and the M2-associated marker CD206 after treatment. (**E**) Phagocytic activity assay after treatment. Reproduced with permission from Ref. [81]. (**F**) qRT-PCR analysis of M1/M2-associated marker expression in a co-culture system. (**G**) Immunofluorescence staining of tumor sections for CD80 and CD206 in vivo. Adapted with permission from Ref. [82]. (**H**) Schematic illustration of the preparation of the exosome-based nanoplatform Exo@MnIO&BG. (**I**) Flow cytometric analysis of M1-type and M2-type macrophages after treatment. Adapted with permission from Ref. [86]

Fe-MNPs can directly leverage their intrinsic physicochemical properties to modulate macrophage polarization. For instance, Horvat et al. developed a core-crosslinked polymeric micelle platform (CCPMs) for SPIONs **(**Fig. 7A**)**. Upon co-treatment with SPION-CCPMs, macrophages exhibited a significant upregulation of the M1-associated surface marker CD86, reaching 1.5-fold higher levels compared to the control group (Fig. 7B), along with increased Nos2 mRNA expression (Fig. 7C). In contrast, expression of the M2-associated marker CD206 was reduced to approximately 50% of the control level (Fig. 7D). Moreover, SPION-CCPM-treated cells showed a 1.8-fold increase in CFSE fluorescence intensity at 24 h (Fig. 7E), indicating enhanced phagocytic activity [81]. Similar immunomodulatory effects have also been reported in clinically relevant iron oxide formulations. Zanganeh et al. demonstrated that ferumoxytol, an FDA-approved iron oxide nanoparticle, robustly promoted M1-associated features both in vitro and in vivo. The treatment upregulated M1-associated markers like TNF-α and CD86 while reducing M2 markers such as CD206 and IL-10 (Fig. 7F, G) [82]. Mechanistically, the intracellular degradation of Fe-MNPs may expand the labile iron pool and promote Fenton-mediated ROS production, which has been associated with NF-κB activation and a shift toward M1-like polarization.

To further amplify this reprogramming efficacy, Fe-MNPs can also be engineered as hybrid platforms. A highly advanced strategy involves doping the Fe-MNP crystal lattice with other immunoregulatory metal ions, such as Mn²⁺. While iron drives Fenton chemistry, Mn²⁺ specifically activates the cGAS-STING pathway, a critical sensor of cytosolic DNA that leads to robust type I interferon (IFN-I) production and subsequent M1-like polarization [83–85]. This highlights one advantage of iron-based platforms: they can integrate the ROS-generating properties of iron with the distinct signaling capabilities of other metals. Wei et al. applied this concept by constructing an exosome platform featuring Mn-doped iron oxide nanoparticles (Exo@MnIO&BG). In a breast cancer model, this system successfully activated the STING pathway and dramatically remodeled the TAM population, reducing M2-type TAMs from 37.2% to 18.4% while increasing M1-type TAMs from 11.9% to 29.8% (Fig. 7H-I) [86]. Additionally, modifying the nanoparticle surface with targeting moieties (e.g., hyaluronic acid) or metabolic enzymes (e.g., lactate oxidase) allows for the simultaneous modulation of multiple immunosuppressive pathways [87, 88].

Despite these robust reprogramming capabilities, the immunological duality of iron metabolism should be acknowledged as a potential limitation. Macrophages are central regulators of systemic and tissue iron homeostasis [89]. Within the TME, M2-like macrophages and many TAM populations often exhibit an iron-release phenotype, thereby increasing local iron availability and potentially supporting tumor cell proliferation and progression [90]. Because Fe-MNPs are readily internalized by macrophages, their downstream effects are highly context-dependent [91]. If Fe-MNP uptake by TAMs fails to generate sufficient pro-inflammatory and oxidative cues to drive effective M1-like repolarization, the added iron pool could instead remain bioavailable within the TME and become tumor-supportive rather than tumor-restrictive [91, 92]. This possibility highlights the importance of precise targeting strategies and careful dose/formulation optimization to ensure that Fe-MNPs favor M1 reprogramming rather than merely increasing iron availability within the TME.

In summary, Fe-MNPs can effectively reprogram TAMs toward the antitumor M1-like phenotype through complementary mechanisms, including intrinsic iron-driven ROS/NF-κB activation, ion doping (e.g., Mn²⁺) to engage the STING pathway, and surface functionalization. However, realizing their full potential requires overcoming the inherent double-edged nature of macrophage iron metabolism, underscoring the need for precise targeting and optimal dosing. At the same time, whether Fe-MNP-induced TAM reprogramming is durable or only transient under in vivo conditions remains insufficiently understood, because many current studies assess macrophage polarization primarily at relatively early post-treatment time points. This multi-pronged, yet carefully balanced approach to reversing TAM-mediated immunosuppression supports the potential of Fe-MNPs as a promising platform for cancer immunotherapy.

### Dendritic cell maturation and antigen presentation

**Fig. 8:**
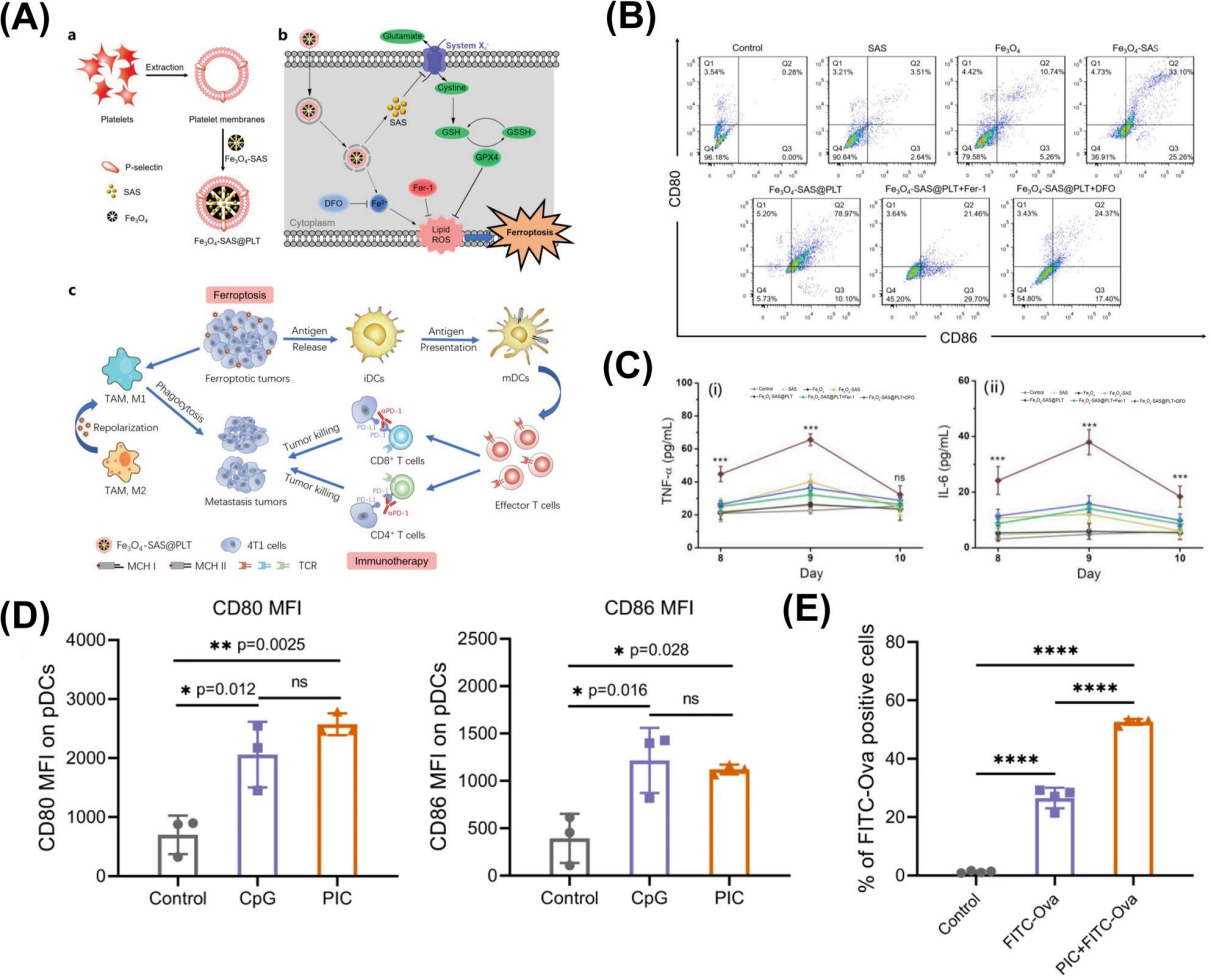
Fe-MNP-induced immunogenic stress promotes dendritic cell maturation and antigen uptake. (**A**) Schematic illustration of the Fe₃O₄-SAS@PLT platform and its ferroptosis-associated immunostimulatory mechanism. (**B**) Flow cytometric analysis of CD80 and CD86 expression on dendritic cells after treatment. (**C**) TNF-α and IL-6 levels after treatment. Reproduced with permission from Ref. [97]. (**D**) CD80 and CD86 mean fluorescence intensity on pDCs after PIC treatment. (**E**) Antigen uptake assay showing the percentage of FITC-Ova-positive cells after PIC treatment. Adapted with permission from Ref. [98]

Dendritic cell (DC) maturation occupies a pivotal position in Fe-MNP-mediated TIME remodeling because it provides a functional bridge between tumor cell stress and adaptive immune activation [93]. Immature DCs are specialized for antigen capture, whereas mature DCs upregulate costimulatory molecules such as CD80 and CD86, enhance antigen presentation, and support the priming of tumor-reactive T cells [94]. Thus, in the context of Fe-MNP-based immunotherapy, the biological significance of DC activation lies not only in phenotypic maturation itself, but also in its capacity to convert local tumor damage into productive antigen-presenting signals [95, 96]. As discussed in Sect. "Primary driving mechanisms of Fe-MNP-mediated immune remodeling", Fe-MNP-mediated thermal, mechanical, and chemical stresses can induce immunogenic tumor cell damage, accompanied by DAMP release, antigen liberation, and regulated forms of cell death. These upstream events create a permissive context for DC activation. For example, Jiang et al. designed a Fe₃O₄-SAS@PLT nanoplatform that triggered ferroptosis-associated tumor stress and was accompanied by marked DC maturation (Fig. 8A). In this system, the proportion of CD80⁺/CD86⁺ activated DCs increased substantially, reaching 78.97% (Fig. 8B), together with elevated systemic levels of proinflammatory cytokines such as TNF-α and IL-6 (Fig. 8C) [97]. These findings support the view that Fe-MNP-induced ferroptotic or immunogenic stress can facilitate the transition of immature DCs into mature antigen-presenting cells.

Moving beyond indirect activation via tumor cell death, Fe-MNP-based platforms can also directly enhance the antigen-handling capacity of DC-associated compartments. Zhang et al. developed a multifunctional nanoparticle platform (PIC) integrating iron oxide with immunostimulatory components, and showed that this system increased CD80 and CD86 mean fluorescence intensity on pDCs (Fig. 8D). More importantly, it directly augmented antigen uptake, as reflected by the significantly higher proportion of FITC-Ova-positive cells after treatment (Fig. 8E) [98]. Although the immune effects of such composite systems cannot be attributed to iron oxide alone, these results reinforce the view that Fe-MNP-based platforms can support the acquisition and processing of tumor-associated antigens, thereby strengthening the basis for downstream T-cell priming. Related Fe-MNP systems have likewise been used to facilitate antigen release, capture, and trafficking to DC-relevant compartments, including dual catalytic oxide nanosponges that retain tumor-associated antigens after magnetic stimulation and RBC-hitchhiking iron oxide nanostructures that deliver captured neoantigens and DAMPs to lymph nodes [95, 96].

DC maturation markers such as CD80 and CD86, although informative, do not by themselves establish durable cross-presentation or long-term T-cell priming [99]. Crucially, the iron-dependent nature of these platforms also introduces a context-dependent immunological risk. While physiological levels of ROS can promote DC activation, excessive iron-driven oxidative stress may impair antigen-processing function or reduce the immunostimulatory capacity of DCs [65], indicating that iron loading and redox stress must be carefully controlled. Therefore, while DC activation represents a major downstream consequence of Fe-MNP-mediated tumor stress, strict dose optimization is required to ensure that iron-driven oxidative stress remains immunostimulatory rather than detrimental to these vital antigen-presenting cells [100]. These DC-centered events nevertheless provide a critical immunological basis for the T-cell responses discussed in the following section.

### T-cell infiltration, activation, and synergy with immunotherapy

T-cell activation represents the most direct functional endpoint of successful Fe-MNP-mediated immune remodeling. Cytotoxic CD8⁺ T cells are the principal effectors of tumor cell killing, whereas CD4⁺ helper T cells support antitumor immunity through cytokine secretion and coordination of other immune compartments. By contrast, regulatory T cells (Tregs) restrain effector T-cell function and, when enriched within tumors, contribute to immune evasion. In this context, hypoxia alleviation, TAM reprogramming, and dendritic cell maturation act in a coordinated manner to establish a more permissive environment for T-cell infiltration and activation.

**Fig. 9:**
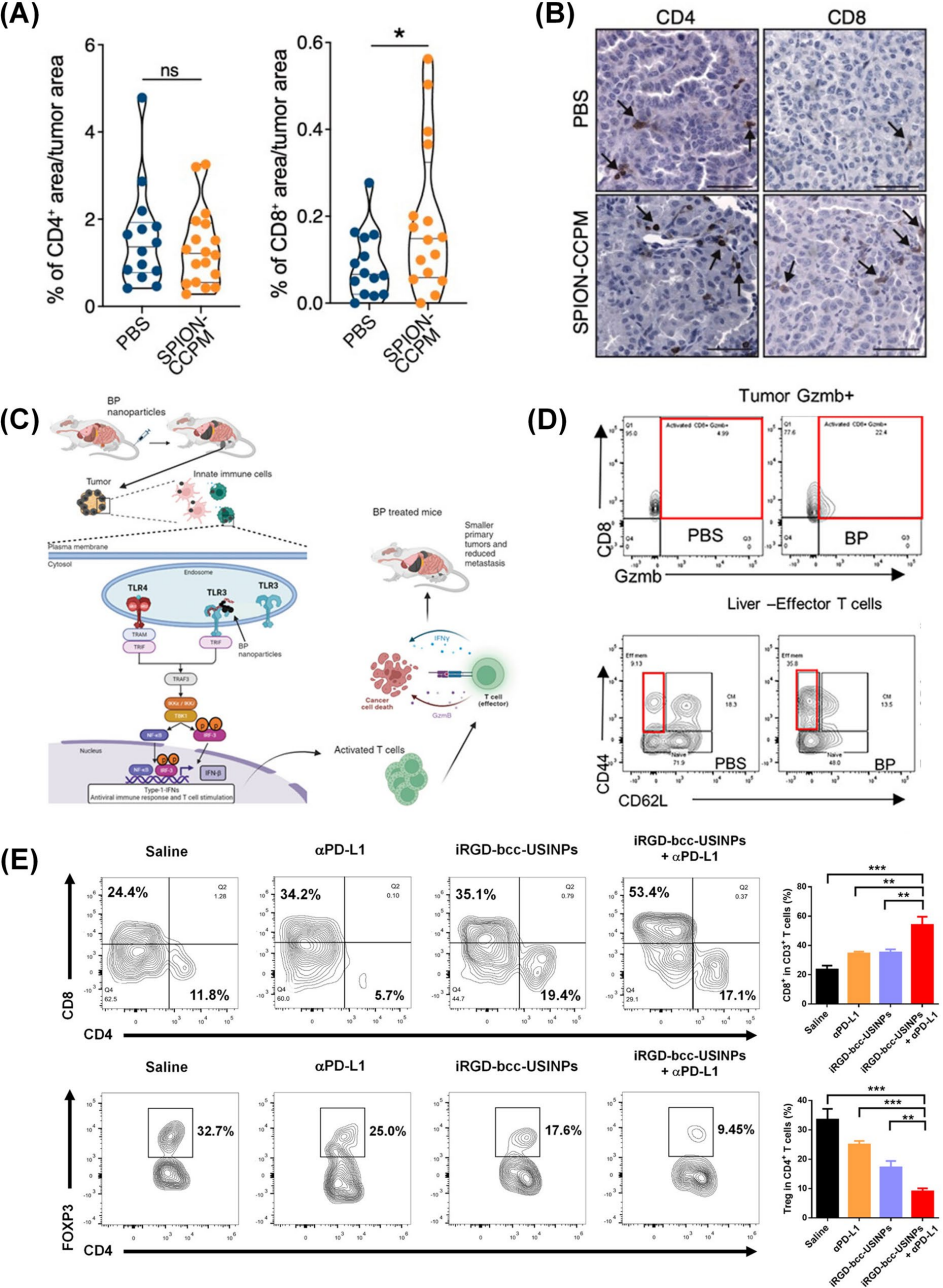
Fe-MNP-mediated T-cell infiltration, activation, and synergy with checkpoint blockade. (**A**) Flow cytometric quantification of intratumoral CD4⁺ and CD8⁺ T-cell areas after SPION-CCPM treatment. (**B**) Immunohistochemical staining of CD4 and CD8 in tumor sections after SPION-CCPM treatment. Reproduced with permission from Ref. [81]. (**C**) Schematic illustration of bionized nanoferrite plain (BP) nanoparticles activating innate immune signaling pathways. (**D**) Flow cytometric analysis of granzyme B-associated CD8⁺ T cells and liver effector T-cell phenotypes after BP treatment. Reproduced with permission from Ref. [101]. (**E**) Flow cytometric analysis of CD8⁺ T cells and Tregs in different treatment groups receiving bcc-USINPs and anti-PD-L1 therapy. Adapted with permission from Ref. [102]

Several Fe-MNP-based systems have been shown to reshape this T-cell landscape. For example, Horvat et al. reported that SPION-CCPM treatment was associated with increased intratumoral CD8⁺ T-cell infiltration, whereas changes in CD4⁺ T-cell abundance were less pronounced (Fig. 9A–B) [81]. These findings are consistent with the view that Fe-MNP-mediated macrophage repolarization and local inflammatory activation can create a niche more favorable for cytotoxic T-cell recruitment. Similarly, Korangath et al. showed that systemic exposure to bionized nanoferrite plain (BP), a starch-coated iron oxide nanoparticle formulation, altered the host immune landscape through TLR-associated innate immune signaling and was accompanied by increased granzyme B-associated CD8⁺ T-cell activity, higher splenic dendritic-cell abundance, and a shift toward more activated effector T-cell phenotypes, together with inhibition of tumor growth and metastasis (Fig. 9C, D) [101]. These results suggest that Fe-MNPs can enhance T-cell immunity not only indirectly, by remodeling upstream immune compartments, but also more broadly, by converting the TIME into a more inflamed and permissive state.

Such T-cell-oriented remodeling is particularly important because it may sensitize tumors to immune checkpoint blockade. Liang et al. demonstrated this using ultrasmall iron nanoparticles (bcc-USINPs), which moderately improved the T-cell landscape on their own but showed substantially greater efficacy when combined with anti-PD-L1 therapy. In the combination group, the proportion of CD8⁺ T cells increased to 53.4%, whereas the Treg fraction declined to 9.45%, clearly exceeding the effects of either monotherapy (Fig. 9E) [102]. These findings support the view that Fe-MNP-mediated TIME remodeling can promote the conversion of immunologically “cold” tumors toward states more responsive to checkpoint inhibition.

However, the promotion of T-cell-mediated immunity by Fe-MNPs must be considered alongside the metabolic vulnerability of activated T cells themselves. Activated CD8⁺ T cells are metabolically vulnerable to lipid peroxidation. Indeed, prior studies have shown that enhanced lipid uptake, such as CD36-associated lipid accumulation in tumor-infiltrating CD8⁺ T cells, can promote lipid peroxidation and ferroptosis while reducing effector cytokine production, including IFN-γ and TNF-α [103]. The enhancement of T-cell responses by Fe-MNPs is often more pronounced in engineered composite systems or in combination with checkpoint blockade, and this effect depends substantially on platform design and therapeutic context.

Overall, current evidence suggests that Fe-MNP-based platforms can promote a more favorable context for T-cell-mediated antitumor immunity by improving upstream conditions such as hypoxia status, macrophage polarization, and DC-associated antigen presentation (Table 1). Nevertheless, further translation will require careful control over the timing, dose, and spatial distribution of Fe-MNP-mediated stimulation to preserve antitumor efficacy while minimizing potential harm to infiltrating T cells.

**Table 1:** Representative studies of Fe-MNPs in cancer immunotherapy

Nanocarrier platform (including surface modification)	Main immunomodulatory mechanism	Combination strategy	Tumor/cell model	Major findings	Ref.
Mitochondria-targeted magnetothermal nanomedicine (MRT; RGD/TPP-functionalized magnetic nanoparticles)	Magnetothermal ICD, DAMP release, and TAM M1 polarization	Magnetic hyperthermia-based immunotherapy	In vitro tumor cells + bilateral murine tumor model	Induced ATP/HSP70 release, promoted M1-like macrophage activation, and inhibited primary and distant tumors	[35]
Ferrimagnetic vortex iron oxide nanorings (FVIOs)	Mild magnetic hyperthermia-induced ICD, macrophage M1 polarization, enhanced T-cell infiltration, and reduced immunosuppressive cells	Mild magnetic hyperthermia + PD-L1 blockade	Orthotopic 4T1 breast cancer model with spontaneous lung metastasis and bilateral distant-tumor (abscopal) setting	Increased CD8⁺ and CD4⁺ T-cell infiltration, reduced Tregs/MDSCs, suppressed primary tumor growth, prevented lung metastasis, and inhibited distant tumor progression	[36]
CD44/CPP-functionalized polyhedral magnetic nanoparticles (PMNPs)	Magneto-mechanical damage and ferroptosis-related stress	Rotating magnetic field (15 Hz)	Gastric cancer stem cells + subcutaneous xenograft	Increased intracellular Fe²⁺, reduced GPX4, enhanced apoptosis/ferroptosis, and suppressed tumor growth	[49]
Acid-responsive Fe/Mn bimetallic MOF nanosystem carrying R848 (FeMn@R@H)	Fenton-mediated ROS generation, NLRP3 activation, pyroptosis, and ICD	TLR7/8 agonist (R848)	4T1 cells + murine tumor model	Triggered pyroptosis and augmented antitumor immune activation	[58]
Mannose-decorated PPS-loaded SPIONs (Man/PPS-SPIONs)	IFN-γ/Fenton/NF-κB/MAPK-driven TAM repolarization	PPS-based immunostimulatory combination	4T1 orthotopic breast cancer model + macrophage system	Increased M1-type TAMs, decreased M2-type TAMs, and improved antitumor efficacy	[59]
Chlorin e6/GOX/HCPT-loaded iron-based MOF (Ce6@HGMOF)	Catalase-like O₂ generation, hypoxia reversal, GSH depletion, ferroptosis induction, and PDT/starvation synergy	Photodynamic therapy + ferroptosis + starvation therapy	EC109 esophageal cancer cells + nude mouse xenograft model	Reversed hypoxia, downregulated HIF-1α/VEGF signaling, induced ferroptosis, and enhanced multimodal antitumor efficacy	[72]
Core-cross-linked polymeric micelle SPIONs (SPION-CCPMs)	TAM repolarization and enhanced CD8⁺ T-cell recruitment	Adjuvant treatment in post-crizotinib setting	ALK⁺ NSCLC-associated macrophage coculture + lung tumor regrowth model	Increased CD86 and Nos2, decreased CD206, enhanced phagocytic activity, and delayed tumor regrowth	[81]
Ferumoxytol	Pro-inflammatory macrophage polarization associated with iron-driven ROS/Fenton activity	None	Tumor-bearing mouse models (MMTV-PyMT breast cancer, SCLC liver/lung metastases)	Upregulated M1 markers, downregulated M2 markers, and inhibited tumor growth/metastasis	[82]
Multifunctional exosome loaded with Mn-doped iron oxide, GW4869, and BSO (Exo@MnIO&BG)	Ferroptosis enhancement, cGAS-STING activation, and TAM remodeling	None	Orthotopic 4T1 breast cancer model with lung metastasis	Enhanced ferroptosis, activated cGAS-STING signaling, reduced M2 TAMs, increased M1 TAMs	[86]
Platelet membrane-camouflaged Fe₃O₄-SAS nanoparticles (Fe₃O₄-SAS@PLT)	Ferroptosis-associated DC maturation, macrophage repolarization, and immune activation	PD-1 blockade	Metastatic 4T1 breast cancer model	Enhanced DC maturation and improved anti-PD-1 therapeutic efficacy with sustained antitumor effects	[97]
Polylysine/iron oxide/CpG nanoparticle (PIC)	In situ vaccination enhancement, increased M1/M2 ratio, type I IFN activation, and improved antigen capture/cross-presentation	Intratumoral PIC + local RT (12 Gy) + systemic anti-CTLA-4	Syngeneic “cold” tumor models (B78 melanoma, MyC-CaP prostate, TCI1 breast)	Potentiated RT-induced in situ vaccination, improved survival, and generated immune memory	[98]
BP starch-coated iron oxide nanoparticles	Systemic innate immune activation via TLR/TRIF/IRF3 signaling and enhanced effector T-cell responses	None (systemic immune nano-adjuvant effect)	Metastatic breast cancer models (MMTV-huHER2 transgenic, Py230 allograft)	Suppressed primary tumor growth and lung metastases, extended survival, and showed immune benefit despite minimal tumor retention	[101]
Renal-clearable ultrasmall single-crystal Fe nanoparticles (iRGD-bcc-USINPs)	Ferroptosis induction, ICD, DC maturation, and adaptive T-cell activation	Anti-PD-L1 immune checkpoint blockade	Syngeneic MC38 and 4T1 tumor models	Potentiated anti-PD-L1 therapy, promoted immune memory, showed strong antitumor efficacy, and retained favorable renal clearance	[102]

## Emerging opportunities of Fe-based magnetic nanoparticles in cancer immunotherapy

### Deep immune intervention based on magnetic force/magnetothermal effects

A key feature that distinguishes Fe-MNPs from non-magnetic iron nanomaterials or other ROS-generating platforms is that magnetism in these systems is not merely a material label, but an enabling property that provides external spatiotemporal control. By integrating magnetic actuation and mild magnetothermal effects within a single platform, Fe-MNPs offer remote, programmable, and potentially image-compatible modes of immune intervention, particularly in deep tissues. Compared with optical or ultrasonic approaches, magnetic fields provide superior tissue penetration and controllability, thereby creating opportunities for immune modulation that extend beyond conventional local stimulus delivery.

(i) ***Magnetically driven immunoregulation***: Under low-frequency dynamic magnetic fields, particularly rotating magnetic fields, Fe-MNPs can generate mechanical perturbations at the level of cellular and subcellular membranes. This non-thermal mode of stimulation may alter membrane tension and permeability, promote DAMP release, and activate inflammatory signaling through mechanosensitive ion channels such as Piezo1 and TRPV, thereby enhancing the DC–T cell axis [104]. A key future challenge is to establish quantitative relationships between magnetic input and immunological output. For example, it remains unclear to what extent specific field strength, frequency, or exposure duration can induce membrane and organelle-level mechanical perturbation, intracellular Ca²⁺ influx, and subsequent immune activation. At the same time, future studies should also clarify how nanoparticle size, shape anisotropy, magnetic moment, and aggregation state jointly influence the efficiency of force transduction, because the fundamental driver of membrane disruption and organelle stress in this context is the magnetic force itself rather than heat alone. By linking such physical parameters to biological readouts, magnetically driven actuation may be further developed into a more predictable sensitizing strategy for immune checkpoint blockade.
(ii) ***Magnetothermal immunological prospects***: Magnetothermal stimulation exerts temperature-dependent immunological effects, and current evidence supports the 41–43 °C range as the most consistently supported window for immune sensitization rather than a universally fixed optimal threshold [105]. At temperatures below 40 °C, heat stress is generally insufficient to induce robust immunogenic responses [106]. In the mild range of approximately 40–41 °C, early heat stress responses begin to emerge, including initial HSP expression, and immune activation may already be detectable in some systems, although these effects are generally less consistent than those observed at 41–43°C [107, 108]. By contrast, within the 41–43 °C interval, HSP70/90 upregulation, ICD-associated signaling, dendritic cell maturation, and antigen presentation have been more consistently associated with hyperthermia [109, 110]. Notably, 43 °C is better regarded as an important reference threshold at which heat cytotoxicity becomes more pronounced, rather than as a universally optimal set point, because the magnitude and quality of the response also depend on exposure duration, heating uniformity, tumor type, and cellular context [111]. As heating extends toward higher-temperature ranges, the balance may shift from immune sensitization toward apoptosis, protein denaturation, and increasing tissue stress, whereas thermal ablation and necrosis become more dominant and the resulting immune consequences less predictable [112, 113]. Accordingly, the future value of Fe-MNP-mediated magnetothermal therapy lies not in defining a single universal temperature threshold, but in establishing a controllable and quantifiable thermal–immune window. Future studies should therefore move beyond nominal temperature reporting and establish standardized magnetothermal dosimetry by clarifying how magnetic input parameters, temperature–time profiles, HSP dynamics, ICD-related markers, and downstream immune outcomes are quantitatively linked.

Beyond defining such a local thermal–immune window, an additional future question is whether magnetothermal stimulation can be used to bridge local stimulation with systemic immune activation. In this regard, the abscopal effect remains an important direction for further investigation, because local hyperthermia-induced DAMP release and HSP upregulation may enhance antigen presentation and systemic T-cell activation, thereby contributing to the suppression of distant untreated lesions [114]. In this context, magnetothermal therapy may serve as a strategy to connect local stimulation with broader immune responses, particularly when combined with immune checkpoint inhibitors or personalized vaccination approaches, although this possibility still requires more rigorous mechanistic and scheduling-based validation.

### Magnetically regulated ferroptosis–immunity synergy

A major future opportunity for Fe-MNPs lies in enabling magnetically regulated ferroptosis–immunity coupling, rather than merely inducing ferroptotic tumor damage. Compared with conventional chemically driven ferroptosis, Fe-MNPs provide a platform in which ferroptosis-associated stress may be modulated not only through iron-dependent redox activity, but also through externally applied magnetic force or mild magnetothermal stimulation. In principle, such magnetic actuation could influence membrane mechanics, organelle stress, and cellular metabolic state, thereby affecting the timing, intensity, and immunogenic consequences of ferroptosis [57]. The central challenge, however, is not simply to enhance ferroptosis, but to regulate its timing and intensity in a way that favors immune activation [64, 65]. In this context, the future value of magnetic guidance lies in whether external magnetic input can be used to confine ferroptosis-associated stress to a more controllable and biologically favorable range. This may provide a practical route to make ferroptosis–immunity coupling more predictable and more therapeutically useful.

Future directions may include: (i) combining GPX4 inhibitors or system Xc⁻ inhibitors with magnetic actuation or mild magnetothermal stimulation to further reduce the ferroptosis threshold [60]. Such combinations may allow lower pharmacological doses to remain effective, thereby reducing systemic toxicity while enabling dual regulation through both biochemical and physical triggers. (ii) establishing monitoring frameworks for ferroptosis-associated immune biomarkers, such as dynamic HMGB1 release, oxidized phospholipid accumulation, or macrophage iron-handling status, in order to evaluate the actual immunological consequences of ferroptosis [89, 90]. Such readouts may provide a basis for adjusting magnetic or thermal input parameters in a more individualized and controllable manner. (iii) clarifying how magnetic input parameters, nanoparticle properties, and ferroptosis-associated immune outputs can be quantitatively linked, so that ferroptosis–immunity coupling can be developed from an empirical phenomenon into a more predictable therapeutic strategy.

### Imaging-guided closed-loop immunoregulation and theranostics

One of the most promising future directions for Fe-MNPs lies in their potential to evolve beyond therapeutic carriers into integrated theranostic platforms that couple imaging, intervention, and feedback control. The future significance of imaging in these systems lies not simply in visualizing nanoparticle distribution, but in enabling imaging-guided adjustment of magnetic intervention. Because Fe-MNPs are intrinsically MRI-responsive, they may support real-time assessment of nanoparticle localization, intratumoral accumulation, and treatment-associated changes through T₂/T₂* signal variation [115, 116]. When combined with MRI thermometry or other imaging-compatible sensing strategies, such readouts may further provide actionable information for iteratively adjusting magnetic or magnetothermal stimulation [117].

This imaging–intervention coupling provides the basis for closed-loop immunoregulation. Unlike conventional monofunctional nanomaterials, Fe-MNPs may enable a more adaptive therapeutic workflow. Nanoparticle localization can first be visualized. Magnetic or magnetothermal stimulation can then be delivered according to distribution patterns. Treatment intensity may subsequently be adjusted based on imaging-defined response. Such a strategy could improve the precision of immune modulation by reducing under-stimulation in poorly reached regions while avoiding excessive stimulation in highly enriched areas. More importantly, imaging can be functionally coupled to magnetically addressable physical stimuli, such as force and heat, together with iron-dependent redox-mediated intervention within a single platform. This feature makes it possible to coordinate magnetic actuation, mild hyperthermia, and ferroptosis-associated immune activation in a temporally ordered and potentially adaptive manner, rather than delivering them as fixed one-step inputs.

From an immunological perspective, this theranostic capability may be especially valuable in heterogeneous tumors. For example, magnetic actuation and mild magnetothermal stimulation may be applied in a staged manner to promote DAMP release, ferroptosis-associated stress, HSP expression, and antigen presentation [118]. In this setting, imaging feedback is valuable not because it directly measures immune activation, but because it helps determine whether the physical and microenvironmental conditions required for effective immune sensitization have been achieved [119]. In this way, the future value of Fe-MNP-based theranostics lies not only in simultaneous diagnosis and treatment, but in enabling feedback-informed and individualized immune modulation. Such convergence of imaging, externally controllable stimulation, and immunoregulatory output is difficult to achieve within a single unified platform.

Future development should therefore focus on establishing reproducible relationships between imaging signals, magnetic dosimetry, and immune outcomes [12, 120]. It will be important to determine how MRI-visible nanoparticle distribution, field input parameters, and downstream readouts such as HSP dynamics, ICD-associated markers, dendritic-cell activation, or T-cell infiltration can be quantitatively integrated into a unified treatment framework. It will also be valuable to explore whether imaging-derived surrogate readouts related to nanoparticle distribution, iron handling, or immune-cell-associated changes can provide practical feedback for treatment adjustment. If such relationships can be standardized, Fe-MNPs may provide a foundation for image-guided and closed-loop cancer immunotherapy with improved precision and biological responsiveness.

## Translational challenges and clinical considerations

### Biosafety, biodegradation, and long-term fate of Fe-MNPs

The clinical translation of Fe-MNPs requires a clear understanding of their biosafety, biodegradation, and long-term fate in vivo. Following systemic administration, Fe-MNPs are predominantly sequestered by the mononuclear phagocyte system (MPS), with major accumulation in the liver, spleen, bone marrow, and other phagocyte-rich tissues [121]. While this distribution pattern may limit acute systemic toxicity, it also raises important concerns regarding long-term organ retention, iron overload, and off-target biological effects.

A key advantage of Fe-MNPs is their potential biodegradability through endogenous iron metabolic pathways. After cellular uptake, Fe-MNPs are trafficked to lysosomes, where acidic pH and enzymatic activity promote gradual degradation of the iron oxide core [122]. The released iron ions may then enter the labile iron pool and become integrated into cellular iron homeostasis pathways, including ferritin storage [123], ferroportin-mediated export followed by extracellular transferrin binding, and subsequent iron reutilization [124, 125]. However, biodegradability does not eliminate risk; rather, it shifts the safety question from permanent particle persistence to whether released iron can be effectively buffered and cleared without disturbing systemic or local iron homeostasis.

Importantly, Fe-MNP biosafety should be viewed as a dose-, time-, and formulation-dependent balance rather than a binary toxic/nontoxic property. High-dose exposure may increase lipid peroxidation and alter hepatic iron-handling or antioxidant-response genes [126, 127], whereas lower-dose or clinically oriented exposure regimens are generally associated with better tolerability [128]. Moreover, clinically relevant iron oxide systems can differ substantially in coating chemistry, colloidal stability, complement activation, immunocompatibility, and hypersensitivity-related potential, underscoring the need for formulation-specific safety evaluation [129]. Existing safety experience from clinically used iron oxide systems remains informative, but still insufficient for direct generalization to newly engineered Fe-MNP platforms designed for cancer immunotherapy.

A related and equally important issue concerns long-term persistence and chronic tissue response. Iron oxide nanoparticles may undergo progressive erosion and biotransformation over time [130], with detectable fractions persisting in the liver and spleen for extended periods [131]. Although many animal studies report acceptable overall tolerability, prolonged retention may still contribute to chronic oxidative stress, low-grade inflammation, or altered tissue microenvironments, particularly under repeated dosing conditions. Accordingly, the key translational question is not simply whether Fe-MNPs appear safe shortly after administration, but whether their degradation products, retention profiles, and iron-associated biological effects remain controllable over time. Future studies should therefore place greater emphasis on long-term biodistribution tracking, biodegradation kinetics, repeated-dose toxicology, and immune safety evaluation under clinically relevant conditions.

### Delivery efficiency, biodistribution, and tumor targeting

Effective tumor delivery remains a central challenge for the clinical translation of Fe-MNPs. Following systemic administration, only a small fraction of nanoparticles typically reaches solid tumors, whereas substantial accumulation occurs in mononuclear phagocyte system (MPS)-rich organs such as the liver and spleen [132]. Meta-analyses indicate that the median tumor delivery efficiency of nanoparticles remains very low, at approximately 0.67–0.7% of the injected dose, with little improvement over the past 15–20 years [133]. Although both passive targeting through the enhanced permeability and retention (EPR) effect and active targeting via ligand functionalization have been extensively explored, their translational performance remains constrained by the heterogeneity of human tumors, including variable vascular permeability, dense stromal and extracellular matrix barriers, and marked intertumoral as well as interpatient variability [134]. Moreover, active targeting does not bypass these barriers, because ligand-mediated interactions can only operate after nanoparticles have successfully extravasated into the tumor [134]. This limitation is illustrated by quantitative analyses showing that fewer than 14 out of 1 million intravenously administered ligand-coated nanoparticles reached target cancer cells, with most retained particles instead localizing to stromal compartments or perivascular phagocytes [135]. This limitation is likely to be even more pronounced in clinical tumors, where dense extracellular matrix, elevated interstitial fluid pressure, poor blood flow, and heterogeneous vascular structure further restrict nanoparticle penetration beyond what is typically observed in conventional xenograft models.

Nevertheless, limited intratumoral accumulation does not necessarily abolish therapeutic benefit [101]. In selected Fe-MNP systems, systemic exposure and uptake in MPS-rich organs have been associated with activation of innate immune pathways and downstream antitumor responses, even when nanoparticle retention within the tumor is limited [101]. A representative example is provided by starch-coated iron oxide nanoparticles, which induced systemic antitumor effects and suppression of lung metastases in breast cancer models despite minimal tumor retention, together with evidence of TLR/TRIF/IRF3-related immune activation [101]. At the same time, such effects appear to be strongly formulation- and context-dependent. For example, recent work shows that distinct surface chemistries on iron oxide nanoparticles can differentially engage complement pathways and phagocyte responses, leading either to antitumor immune activation or to less favorable immune outcomes [136]. Accordingly, these observations should not be generalized as a substitute for efficient tumor targeting. Rather, they suggest that the biological consequences of Fe-MNP biodistribution are more complex than a simple “high tumor accumulation = success, off-tumor uptake = failure” framework.

Strategies to improve biodistribution, including biomimetic surface engineering and rational optimization of particle size, shape, and surface chemistry, may prolong circulation and improve tumor access in selected settings [137]. However, these approaches do not uniformly confer disease-site specificity and often introduce additional challenges in reproducibility, stability, and scale-up. Moreover, even when Fe-MNPs accumulate within tumors, their spatial distribution is frequently heterogeneous, often remaining concentrated in perivascular or stromal regions rather than penetrating deeply into poorly perfused or matrix-dense tumor compartments [135, 138]. This point is supported by spatial analyses showing that even antibody-conjugated iron oxide nanoparticles retained in tumors were more strongly associated with stromal host cells than with antigen-positive cancer cells [138]. More broadly, dense extracellular matrix, elevated interstitial fluid pressure, heterogeneous vascular permeability, and competitive uptake by stromal or phagocytic cells can all restrict biologically meaningful access to target cells. Thus, bulk tumor accumulation may substantially overestimate target-cell-accessible and functionally relevant intratumoral delivery.

From a translational perspective, Fe-MNP delivery should therefore be evaluated not only by the fraction reaching tumors, but also by whether biodistribution yields therapeutically meaningful effects with acceptable off-target burden, clearance characteristics, and reproducibility [139]. This will require more realistic preclinical evaluation frameworks. Subcutaneous xenografts remain useful for proof-of-concept studies, but they often fail to capture the stromal, spatial, and immune complexity of clinical tumors [140]. Orthotopic and metastatic models provide improved disease-site and dissemination realism, whereas patient-derived xenograft models better preserve intertumoral heterogeneity but are limited for evaluating immunotherapies because they are commonly established in immunocompromised hosts [141]. For Fe-MNP-based cancer immunotherapy, immunocompetent systems and, where feasible, genetically engineered models are therefore likely to be especially important for clarifying how tumor deposition, systemic clearance, and immune engagement interact under clinically relevant conditions [142, 143]. In this context, the translational objective is not merely to maximize tumor uptake as an isolated metric, but to optimize biodistribution in a manner that yields reproducible therapeutic benefit with acceptable translational feasibility.

### Tumor heterogeneity, response variability, and translational considerations

Therapeutic outcomes of Fe-MNP-based cancer immunotherapy are unlikely to be consistent across tumor types, model systems, or treatment regimens. The current literature should therefore be viewed not as a homogeneous body of evidence, but as a heterogeneous and still-evolving preclinical landscape in which tumor-intrinsic biology, host immune context, and experimental design can all shape nanoparticle delivery, immune engagement, and treatment outcome [140, 144, 145]. Accordingly, translational claims should be calibrated to the strength, relevance, and biological context of the supporting evidence.

A major source of variability lies in tumor context. The mechanisms attributed to Fe-MNPs are unlikely to operate with equal relevance across all cancers, because the biological conditions required for hypoxia modulation, TAM reprogramming, ferroptosis induction, or T-cell activation differ substantially among tumor types [12]. In immunologically “cold” tumors with dense stroma, limited baseline T-cell infiltration, and abundant suppressive myeloid cells, Fe-MNPs may first need to shift the tumor microenvironment toward a more inflamed state before meaningful T-cell-mediated immunity can emerge [9]. By contrast, in “hot” tumors with pre-existing immune infiltration, Fe-MNP-induced immunogenic stress may more readily amplify ongoing antitumor responses, although it may also encounter stronger adaptive immune resistance [9, 146]. Likewise, tumors enriched in M2-like macrophages [147], pronounced hypoxia, or iron-dependent vulnerabilities may be more amenable to different Fe-MNP-based intervention strategies, including TAM-directed, oxygen-modulating, or ferroptosis-oriented approaches [30]. Taken together, these observations suggest that Fe-MNP efficacy is shaped by tumor-intrinsic biology rather than governed by a single universal mechanism.

This context dependence also makes mechanistic synergy difficult to resolve quantitatively. Fe-MNPs are frequently described as remodeling the tumor immune microenvironment through multiple interconnected processes, including hypoxia alleviation, macrophage reprogramming, ferroptotic or other immunogenic stress, dendritic-cell activation, and enhanced T-cell infiltration [148]. Yet the relative contribution of these mechanisms has not been rigorously quantified within unified experimental frameworks, as the available evidence is largely derived from separate studies using different formulations, tumor models, treatment conditions, and endpoints [149]. Under these circumstances, “synergy” is better regarded as a mechanistic working model than as a quantitatively resolved hierarchy of effects. This limitation is reinforced by the current evidence hierarchy: much of the Fe-MNP literature remains concentrated in in vitro systems and subcutaneous tumor models, whereas orthotopic, metastatic, immunocompetent, patient-derived, and genetically engineered models remain relatively underused [150]. To reduce overinterpretation and better distinguish exploratory findings from more advanced preclinical evidence, representative primary studies were further stratified by tumor context, model type, immune context, safety evaluation, and overall evidence tier (Table 2). Overall, the present evidence base for Fe-MNP immunotherapy remains predominantly early-stage preclinical, and broad translational claims should be made with caution [139, 140].

**Table 2:** Evidence hierarchy of representative Fe-MNP studies for cancer immunotherapy

Representative platform	Model type	Immune context	Safety evaluation	Evidence tier	Main translational limitation		Ref.
MFH	In vitro only	None	In vitro cytocompatibility only	E1	No in vivo validation, no immune context		[34]
MRT	In vitro studies + bilateral murine tumor model	Murine immune context present	Short-term safety only	E3	Immune readouts mainly macrophage-centered, but safety depth remains limited		[35]
FVIO nanorings	Orthotopic syngeneic + bilateral/abscopal model	Immunocompetent	Short-term body weight and histology	E3	Strong immune evidence, but no extended safety or schedule optimization		[36]
CD44/CPP-PMNPs	In vitro CSC model + subcutaneous in vivo tumor model	Limited/not immune-focused	Basic in vivo biocompatibility	E2	Good efficacy signal, but immune context remains limited		[49]
FeMn@R@H MOF	In vitro studies + subcutaneous syngeneic tumor model	Immunocompetent	Body weight, histology, blood biochemistry (short-term)	E3	Still limited to subcutaneous modeling and a complex formulation		[58]
Man/PPS-SPIONs	Orthotopic syngeneic + macrophage studies	Immunocompetent	Limited explicit safety reporting	E3	Strong TME relevance, but safety depth remains limited		[59]
SPION-CCPMs	Genetically relevant lung cancer mouse model	Immunocompetent	Minimal safety reporting	E4	Strong model relevance, but local pulmonary delivery may limit generalization	[81]	
Ferumoxytol	In vitro co-culture + syngeneic/metastatic in vivo models	Mixed (immunocompetent and immunodeficient)	Safety not a primary endpoint	E3	Translationally important formulation, but safety and immune analysis are not comprehensive	[82]	
Exo@MnIO&BG	Orthotopic + metastatic in vivo model	Immunocompetent (implied by model)	Short-term body weight, histology & blood routine	E4	Advanced efficacy model, but platform complexity is high	[86]	
Fe₃O₄-SAS@PLT	Experimental metastatic model + immune assays	Immunocompetent	Limited long-term safety information	E3	Strong combination evidence, but safety depth remains limited	[97]	
PIC	Multiple syngeneic models including orthotopic breast model	Immunocompetent	Safety not a primary endpoint	E4	Strong immune efficacy, but multimodal design complicates attribution to Fe-MNP alone	[98]	
BP IONPs	Syngeneic metastatic models + TRIF knockout mechanistic model	Immunocompetent	Safety not deeply profiled	E4	Strong immune-priming realism, but formulation-specific and still preclinical	[101]	
iRGD-bcc-USINPs	Syngeneic tumor models	Immunocompetent	Hematology, biochemistry, histology, renal clearance	E4	Strong preclinical package, but still short of long-horizon translational validation	[102]	

E1, exploratory mechanistic evidence; E2, supportive but model-limited preclinical evidence; E3, translationally informative preclinical evidence; E4, advanced preclinical evidence. Main translational limitation indicates the key factor limiting direct clinical extrapolation

These limitations become particularly important in combination settings. Fe-MNPs should not be assumed to combine with checkpoint blockade, chemotherapy, or radiotherapy in a simply additive manner, because outcome is likely to depend on dose, timing, sequence, and delivery in relation to the evolving tumor-immune microenvironment and the cancer-immunity cycle [148, 151]. Insufficient exposure may fail to generate adequate tumor priming, antigen release, or macrophage reprogramming, whereas excessive or poorly controlled exposure may increase off-target inflammation, oxidative stress, or immune-related toxicity. In this context, a priming-first strategy—where Fe-MNPs are used to induce immunogenic stress, promote antigen release, activate innate immunity, or facilitate T-cell recruitment before PD-1/PD-L1 blockade—may be preferable to immediate co-administration when checkpoint inhibition alone is unlikely to be sufficient [151]. Even so, schedule-dependent effects in Fe-MNP-based combination therapy remain insufficiently defined, and systematic comparisons of dose, timing, and sequence are still uncommon.

Taken together, these considerations argue against a one-size-fits-all approach to Fe-MNP immunotherapy. Instead, patient stratification and predictive biomarkers will probably be necessary for effective translation. Broader clinical literature indicates that the immune contexture of the tumor microenvironment-particularly pre-existing cytotoxic T-cell infiltration and stromal or vascular features that regulate immune-cell trafficking-has prognostic and theranostic relevance across solid tumors [152, 153]. Potentially informative variables for Fe-MNP-based strategies may include baseline hypoxia, tumor-associated macrophage composition or polarization state, ferroptosis-related iron metabolism signatures, and pre-existing immune infiltration. Although these factors have not yet been validated as Fe-MNP-specific biomarkers, they may help prioritize tumors that are biologically more compatible with distinct Fe-MNP strategies or with combination checkpoint blockade [147, 154]. In addition, clinically approved ferumoxytol formulations may provide a practical imaging-informed reference for future Fe-MNP-guided stratification strategies, particularly in relation to biodistribution assessment and clinical monitoring [155]. More broadly, progress in this field will depend on moving beyond isolated proof-of-concept studies toward better integration of tumor biology, model relevance, dose optimization, treatment scheduling, and biomarker-guided patient selection.

### Manufacturing, standardization, and clinical translation considerations

A realistic discussion of Fe-MNP translation should begin with existing clinical and near-clinical reference points. Iron-based magnetic nanoparticles have already established a limited but meaningful clinical footprint in several areas, including MRI contrast enhancement, iron replacement, sentinel lymph node mapping, and localized magnetic hyperthermia. Representative examples include ferumoxytol, ferumoxides/Feridex, ferucarbotran/Resovist, and Magtrace. NanoTherm is also relevant as a magnetic hyperthermia platform with prior glioblastoma-focused clinical implementation and trial experience [156, 157]. These examples confirm that iron-based nanoplatforms can reach clinical testing and, in selected settings, clinical implementation. At the same time, these examples should be viewed as translational reference points rather than direct proof for multifunctional Fe-MNP immunotherapy. In most cases, their clinical success has relied on narrow indications, relatively simple formulations, and clearly defined clinical workflows, rather than on broad immune-remodeling claims [139]. More importantly, these examples highlight a sobering point for the field. The clinical value of nanomedicine often depends less on dramatic gains in tumor-selective delivery and more on improved pharmacokinetics, reduced toxicity, or better compatibility with existing therapy. Thirty years of clinical experience with pegylated liposomal doxorubicin illustrate this well, as its most durable asset has been reduced cardiotoxicity rather than uniformly superior efficacy [158, 159]. For Fe-MNPs, the key translational question is therefore not how many functions can be integrated into one platform, but whether a formulation solves a clinically meaningful problem better than current standard care.

Once that product rationale is defined, manufacturing and quality control become the next gate for translation, and they cannot be separated from long-term safety. As noted in a recent study, nanomedicine failure often arises because manufacturability, stability, and quality control are considered too late; accordingly, early definition of the target product profile, early identification of critical material attributes, critical process parameters, and critical quality attributes, early characterization across multiple unique batches, and early design within an industrial framework are recommended [139]. These requirements are particularly relevant to Fe-MNP systems incorporating membranes, ligands, multiple payloads, or magnetically responsive modules, where minor variations in source materials or processing conditions may alter physicochemical properties, biodistribution, immune activity, and safety. Recent IONP translational reviews further emphasize that repeated-dose toxicology, delayed clearance, and long-term liver/spleen accumulation remain unresolved issues for broader clinical adoption [156]. Even for an established product such as ferumoxytol, current prescribing information retains a boxed warning for serious hypersensitivity/anaphylaxis reactions, requires slow infusion and post-infusion monitoring, and specifically warns about iron overload and MRI interference [160]. In practical terms, translationally serious Fe-MNP development should therefore prioritize simplified formulations, robust analytical methods, defined release specifications, storage stability studies, batch-to-batch reproducibility, and clinically relevant long-term safety assessment.

Beyond product manufacturing and safety, the technical reliability of the intervention itself must also be established when magnetic functionality is involved. Magnetic hyperthermia provides a useful cautionary example. Clinical and technical reviews indicate that therapeutic heating is not defined simply by the presence of magnetic nanoparticles, but by the quality of heating equipment, thermal dosimetry, treatment planning, and treatment control [161]. In glioma-directed magnetic hyperthermia, treatment feasibility and likely effectiveness depend on several factors. These include image-guided planning, intralesional nanoparticle distribution, delivery technique, and AMF parameter selection [157]. For Fe-MNP immunotherapy, this implies that magnetic function should not be presented as a generic multifunctional advantage; it becomes translationally meaningful only when linked to a concrete clinical need and an executable treatment protocol [156].

With these product- and technology-level requirements in place, clinical implementation depends on patient selection and trial design. As highlighted in the literature, target-population definition, biomarker-backed clinical design, and patient stratification should be considered high-priority translational requirements [139]. This is especially relevant for Fe-MNPs because interpatient variability in nanoparticle accessibility remains substantial, and because generic EPR assumptions are unreliable in clinical tumors. Future trials should therefore not simply enroll unselected populations and assume that multifunctionality will compensate for biological heterogeneity. Instead, they should enrich for tumors with relevant immune, stromal, iron-metabolic, or biodistribution features, and define endpoints that reflect the actual advantage of the platform [156, 158]. In many settings, a realistic objective may be non-inferior efficacy with lower toxicity or superior combinability, rather than large superiority margins over standard-of-care therapy in unstratified cohorts.

Several research priorities emerge from the current evidence base. One is the need for unified experimental systems. These should clarify how magnetic hyperthermia, magneto-mechanical perturbation, iron-dependent oxidative stress, and downstream immune remodeling interact within the same Fe-MNP platform. Another is the need to focus on several biological questions that remain unresolved. These include how ferroptosis-related stress links to immune activation, whether TAM reprogramming is durable, and how hypoxia modulation affects immune permissiveness. More standardized frameworks are also needed for magnetic dosimetry, imaging–intervention correlation, immune endpoint assessment, and long-term safety evaluation. Together, these priorities may help move the field from descriptive proof-of-concept studies toward more mechanistically resolved and clinically actionable development.

Taken together, the translational roadmap for Fe-MNP-based cancer immunotherapy should become more selective, more indication-driven, and less formulation-centric. The most clinically relevant next-generation platforms will probably not be the most complex ones, but those that combine a manufacturable design, an acceptable long-term safety profile, a technically executable treatment strategy, a biomarker-informed patient population, and a feasible clinical workflow. For this field, depth of translational planning will matter more than breadth of functionality.

## Conclusions

In summary, iron-based magnetic nanoparticles (Fe-MNPs) represent a distinctive class of multifunctional nanoplatforms that can actively remodel the tumor immune microenvironment rather than merely act as passive carriers. By integrating magnetic responsiveness with iron-dependent redox activity, Fe-MNPs are able to generate coordinated thermal, mechanical, and chemical stimuli, which converge to induce immunogenic stress and regulated tumor cell injury, and subsequently reshape both innate and adaptive immunity. Within this unified framework, upstream physicochemical perturbations can be translated into hypoxia relief, tumor-associated macrophage reprogramming, dendritic cell activation, and enhanced T-cell infiltration, thereby providing a coherent mechanistic basis for the emerging role of Fe-MNPs in cancer immunotherapy. These features position Fe-MNPs as promising platforms for magnetically regulated, imaging-compatible, and immunologically active cancer intervention.

At the same time, the current evidence base remains predominantly preclinical, and several key issues must be resolved before Fe-MNPs can move toward clinically meaningful application. In particular, future progress will depend on clarifying the relative contribution and dose dependence of different mechanisms in unified experimental systems, improving the clinical relevance of model selection, and establishing reproducible links among nanoparticle properties, magnetic input, biological response, and therapeutic outcome. Equally important are the translational requirements of long-term biosafety, biodegradation, delivery realism, manufacturing standardization, and biomarker-guided patient selection. Overall, the future of Fe-MNP-based cancer immunotherapy will likely depend less on further increasing platform complexity and more on developing mechanism-informed, standardized, and clinically executable strategies that align material design with realistic therapeutic needs.

## Data Availability

No datasets were generated or analysed during the current study.

## Abbreviations

TIME: Tumor immune microenvironment
Fe-MNPs: Iron-based magnetic nanoparticles
TAMs: Tumor-associated macrophages
TME: Tumor microenvironment
DCs: Dendritic cells
MDSCs: Myeloid-derived suppressor cells
CAFs: Cancer-associated fibroblasts
Tregs: Regulatory T cells
CAR-T: Chimeric antigen receptor T
NK cell: Natural killer cell
PD-1: Programmed death 1
PD-L1: Programmed death-ligand 1
MRI: Magnetic resonance imaging
MHT: Magnetic hyperthermia
DAMPs: Damage-associated molecular patterns
AMF: Alternating magnetic field
MFH: Magnetic fluid hyperthermia
HWH: Hot water hyperthermia
MRT: Magnetic hyperthermia system
ICD: Immunogenic cell death
CTLs: Cytotoxic T lymphocytes
FVIOs: Ferrimagnetic vortex iron oxide nanoparticles
PTT: Photothermal therapy
ICIs: Immune checkpoint inhibitors
ROS: Reactive oxygen species
CLSM: Confocal laser scanning microscopy
EGF: Epidermal growth factor
LAMP1: Lysosomal-associated membrane protein 1
LMP: Lysosomal membrane permeability
CRT: Calreticulin
PMNPs: Polyhedral magnetic nanoparticles
NFAT: Nuclear factor of activated T-cells
CDT: Chemodynamic therapy
H₂O₂: Hydrogen peroxide
•OH: Hydroxyl radicals
GPX4: Glutathione peroxidase 4
HMGB1: High-mobility group box 1
MHC-I: Major histocompatibility complex class I
CAT-like: Catalase-like
Cat-IONP: Catalase-iron oxide nanoparticle composite
PFCs: Perfluorocarbons
SPIO: Superparamagnetic iron oxide nanoparticles
PFH: Perfluorohexane
CHC: Carboxymethyl hexanoyl chitosan
TNF-α: Tumor necrosis factor-alpha
IL-12: Interleukin-12
TGF-β: Transforming growth factor-beta
VEGF: Vascular endothelial growth factor
IFN-I: Type I interferon
LPOs: Lipid peroxides
CSCs: Cancer stem cells
Th cells: Helper T cells
TLR: Toll-like receptor
ICB: Immune checkpoint blockade
Piezo1: Piezo-type mechanosensitive ion channel component 1
TRPV: Transient Receptor Potential Vanilloid channels
HSP: Heat Shock Protein
HSP70/90: Heat Shock Protein 70 and Heat Shock Protein 90
ecto-CRT: Ectopic calreticulin
GMP: Good manufacturing practice

## References

[1] Kloosterman DJ, et al. Macrophages at the interface of the co-evolving cancer ecosystem. Cell. 2023;186(8):1627–51. 10.1016/j.cell.2023.02.020. 36924769 10.1016/j.cell.2023.02.020

[2] de Visser KE, et al. The evolving tumor microenvironment: from cancer initiation to metastatic outgrowth. Cancer Cell. 2023;41(3):374–403. 10.1016/j.ccell.2023.02.016. 36917948 10.1016/j.ccell.2023.02.016

[3] Tufail M, et al. Immune evasion in cancer: mechanisms and cutting-edge therapeutic approaches. Signal Transduct Target Ther. 2025;10(1):227. 10.1038/s41392-025-02280-1. 40739089 10.1038/s41392-025-02280-1 PMC12311175

[4] Lasser SA, et al. Myeloid-derived suppressor cells in cancer and cancer therapy. Nat Rev Clin Oncol. 2024;21(2):147–64. 10.1038/s41571-023-00846-y. 38191922 10.1038/s41571-023-00846-y

[5] Oliveira M, et al. High-definition spatial transcriptomic profiling of immune cell populations in colorectal cancer. Nat Genet. 2025;57(6):1512–23. 10.1038/s41588-025-02193-3. 40473992 10.1038/s41588-025-02193-3 PMC12165841

[6] Wang M, et al. Unraveling temporal and spatial biomarkers of epithelial-mesenchymal transition in colorectal cancer: insights into the crucial role of immunosuppressive cells. J Transl Med. 2023;21(1):794. 10.1186/s12967-023-04600-x. 37940972 10.1186/s12967-023-04600-x PMC10633927

[7] Lv B, et al. Immunotherapy: reshape the tumor immune microenvironment. Front Immunol. 2022;13:844142. 10.3389/fimmu.2022.844142. 35874717 10.3389/fimmu.2022.844142 PMC9299092

[8] Ren T, et al. Mechanical forces and immune cells in the tumor microenvironment: from regulation mechanisms to therapeutic strategies. Int J Surg. 2025;111(8):5420–34. 10.1097/js9.0000000000002636. 40478969 10.1097/JS9.0000000000002636

[9] Wu B, et al. Cold and hot tumors: from molecular mechanisms to targeted therapy. Signal Transduct Target Ther. 2024;9(1):274. 10.1038/s41392-024-01979-x. 39420203 10.1038/s41392-024-01979-x PMC11491057

[10] Sharma P, et al. Immune checkpoint therapy—current perspectives and future directions. Cell. 2023;186(8):1652–69. 10.1016/j.cell.2023.03.006. 37059068 10.1016/j.cell.2023.03.006

[11] Ibrahim A, et al. MDSC checkpoint blockade therapy: a new breakthrough point overcoming immunosuppression in cancer immunotherapy. Cancer Gene Ther. 2025;32(4):371–92. 10.1038/s41417-025-00886-9. 40140724 10.1038/s41417-025-00886-9 PMC11976280

[12] Yang C, et al. Engineered iron oxide nanoplatforms: reprogramming immunosuppressive niches for precision cancer theranostics. Mol Cancer. 2025;24(1):225. 10.1186/s12943-025-02443-2. 40887613 10.1186/s12943-025-02443-2 PMC12400778

[13] Ko MJ, et al. Magnetic nanoparticles for ferroptosis cancer therapy with diagnostic imaging. Bioact Mater. 2024;32:66–97. 10.1016/j.bioactmat.2023.09.015. 37822917 10.1016/j.bioactmat.2023.09.015 PMC10562133

[14] Hsiao C-H, et al. Nano-orchestrated magnetotactic-like navigation for electromagnetic theranostics and immune enhancement via photoautotrophic oxygenation, mild hyperthermia, and ferroptosis. J Nanobiotechnol. 2025;23(1):442. 10.1186/s12951-025-03488-7. 10.1186/s12951-025-03488-7 PMC12164064 40514659

[15] Qiao R, et al. Magnetic iron oxide nanoparticles for brain imaging and drug delivery. Adv Drug Deliv Rev. 2023;197:114822. 10.1016/j.addr.2023.114822. 37086918 10.1016/j.addr.2023.114822

[16] Ghazi R, et al. Iron oxide based magnetic nanoparticles for hyperthermia, MRI and drug delivery applications: a review. RSC Adv. 2025;15(15):11587–616. 10.1039/d5ra00728c. 40230636 10.1039/d5ra00728c PMC11995399

[17] Shakeri-Zadeh A, et al. Imaging-guided precision hyperthermia with magnetic nanoparticles. Nat Rev Bioeng. 2025;3(3):245–60. 10.1038/s44222-024-00257-3. 40260131 10.1038/s44222-024-00257-3 PMC12011369

[18] Li Y, et al. Dynamic magneto-mechanical force in lysosomes induces durable macrophage repolarization for antitumor immunity. Cell Res. 2026. 10.1038/s41422-025-01217-1. 41629559 10.1038/s41422-025-01217-1 PMC12909937

[19] Wang Z, et al. Nanotechnology for tumor ferroptosis. Cell Biomaterials. 2026;2(3):100222. 10.1016/j.celbio.2025.100222.

[20] Li Z, et al. Designing smart iron oxide nanoparticles for MR imaging of tumors. Chemical & Biomedical Imaging. 2023;1(4):315–39. 10.1021/cbmi.3c00026. 37501794 10.1021/cbmi.3c00026 PMC10369497

[21] Anand S, et al. Embracing remote fields as the fourth dimension of tissue biofabrication. Adv Funct Mater. 2024;34(32):2401654. 10.1002/adfm.202401654.

[22] Yao C, et al. Magneto-mechanical therapeutic effects and associated cell death pathways of magnetic nanocomposites with distinct geometries. Acta Biomater. 2023;161:238–49. 10.1016/j.actbio.2023.02.033. 36858162 10.1016/j.actbio.2023.02.033

[23] Yang J, et al. A hollow mesoporous iron oxide nanoparticle to strengthen Fenton reaction and weaken antioxidant defense systems for high efficacy tumor ferroptosis therapy. Chem Eng J. 2024;497:154470. 10.1016/j.cej.2024.154470.

[24] Catanzaro E, et al. Immunogenicity of ferroptosis in cancer: a matter of context? Trends Cancer. 2024;10(5):407–16. 10.1016/j.trecan.2024.01.013. 38368244 10.1016/j.trecan.2024.01.013

[25] Wahida A, et al. Decoding ferroptosis for cancer therapy. Nat Rev Cancer. 2025;25(12):910–24. 10.1038/s41568-025-00864-1. 41073537 10.1038/s41568-025-00864-1

[26] Chen R, et al. DAMPs in the immunogenicity of cell death. Mol Cell. 2025;85(20):3874–89. 10.1016/j.molcel.2025.09.007. 41106375 10.1016/j.molcel.2025.09.007

[27] Wang L, et al. Iron-based nanomaterials for modulating tumor microenvironment. Wiley Interdiscip Rev Nanomed Nanobiotechnol. 2025;17(1):e70001. 10.1002/wnan.70001. 39788569 10.1002/wnan.70001

[28] Gan L, et al. A Fe(2)O(3)/CNx cascade nanoreactor with dual-enzyme-mimetic activities for cancer hypoxia relief to amplify chemo/photodynamic therapy. Colloids Surf B Biointerfaces. 2024;241:114070. 10.1016/j.colsurfb.2024.114070. 38968858 10.1016/j.colsurfb.2024.114070

[29] Del Prete A, et al. Dendritic cell subsets in cancer immunity and tumor antigen sensing. Cell Mol Immunol. 2023;20(5):432–47. 10.1038/s41423-023-00990-6. 36949244 10.1038/s41423-023-00990-6 PMC10203372

[30] Xiang S, et al. Breaking hypoxic barrier: oxygen-supplied nanomaterials for enhanced T cell-mediated tumor immunotherapy. Int J Pharm X. 2025;10:100400. 10.1016/j.ijpx.2025.100400. 41048631 10.1016/j.ijpx.2025.100400 PMC12495343

[31] Zhang K, et al. Macrophage-derived chemokines in T cell regulation: implications for cancer immunotherapy. Front Immunol. 2026;16:1739154. 10.3389/fimmu.2025.1739154. 41635851 10.3389/fimmu.2025.1739154 PMC12861892

[32] Yan B, et al. Reversal of HMGA1-mediated immunosuppression synergizes with immunogenic magnetothermodynamic for improved hepatocellular carcinoma therapy. ACS Nano. 2023;17(10):9209–23. 10.1021/acsnano.3c00004. 37162457 10.1021/acsnano.3c00004

[33] Li M, et al. Magnetothermal-activated gene editing strategy for enhanced tumor cell apoptosis. J Nanobiotechnol. 2024;22(1):450. 10.1186/s12951-024-02734-8. 10.1186/s12951-024-02734-8 PMC11287911 39080645

[34] Rodríguez-Luccioni HL, et al. Enhanced reduction in cell viability by hyperthermia induced by magnetic nanoparticles. Int J Nanomedicine. 2011;6:373–80. 10.2147/ijn.S14613. 21499427 10.2147/IJN.S14613 PMC3075903

[35] Jiang H, et al. Evoking tumor associated macrophages by mitochondria-targeted magnetothermal immunogenic cell death for cancer immunotherapy. Biomaterials. 2022;289:121799. 10.1016/j.biomaterials.2022.121799. 36152515 10.1016/j.biomaterials.2022.121799

[36] Liu X, et al. Ferrimagnetic vortex nanoring-mediated mild magnetic hyperthermia imparts potent immunological effect for treating cancer metastasis. ACS Nano. 2019;13(8):8811–25. 10.1021/acsnano.9b01979. 31328922 10.1021/acsnano.9b01979

[37] Hu A, et al. Controlled intracellular aggregation of magnetic particles improves permeation and retention for magnetic hyperthermia promotion and immune activation. Theranostics. 2023;13(4):1454–69. 10.7150/thno.80821. 36923543 10.7150/thno.80821 PMC10008738

[38] Kaneko M, et al. All-in-one magnetic nanoparticles for thermo-immunotherapy of malignant melanoma. Adv Healthc Mater. 2025;14(20):e2500260. 10.1002/adhm.202500260. 40033977 10.1002/adhm.202500260

[39] Zha M, et al. Repurposing clinical iron oxide agents for mild hyperthermia-assisted cancer therapy. Cell Rep Phys Sci. 2025;6(12):102977. 10.1016/j.xcrp.2025.102977.

[40] Vaupel P, et al. From localized mild hyperthermia to improved tumor oxygenation: physiological mechanisms critically involved in oncologic thermo-radio-immunotherapy. Cancers (Basel). 2023;15(5):1394. 10.3390/cancers15051394. 36900190 10.3390/cancers15051394 PMC10000497

[41] Li B, et al. Sublethal heat stress synergizes with the tumor microenvironment to drive recurrence of hepatocellular carcinoma after thermal ablation: mechanisms, molecular predictors, and targeted interventions. Cell Biol Toxicol. 2026. 10.1007/s10565-026-10160-9. 41661351 10.1007/s10565-026-10160-9 PMC12935749

[42] Mi Y, et al. Decaying oscillating pulsed magnetic field induces lysosome-dependent cell death in A375 melanoma via magneto-mechanical force. Magnetochemistry. 2025;11(4):33. 10.3390/magnetochemistry11040033.

[43] Lee D, et al. Zinc ferrite nanoparticle with enhanced magnetic attraction for controlled drug delivery and in vivo tumor therapy. Chem Eng J. 2025;503:158311. 10.1016/j.cej.2024.158311.

[44] Patrick PS, et al. Improved tumour delivery of iron oxide nanoparticles for magnetic hyperthermia therapy of melanoma via ultrasound guidance and 111In SPECT quantification. Nanoscale. 2024;16(42):19715–29. 10.1039/D4NR00240G. 39044561 10.1039/d4nr00240g PMC11488578

[45] Shen Y, et al. Elongated nanoparticle aggregates in cancer cells for mechanical destruction with low frequency rotating magnetic field. Theranostics. 2017;7(6):1735–48. 10.7150/thno.18352. 28529648 10.7150/thno.18352 PMC5436524

[46] Zhang E, et al. Dynamic magnetic fields remote-control apoptosis via nanoparticle rotation. ACS Nano. 2014;8(4):3192–201. 10.1021/nn406302j. 24597847 10.1021/nn406302j PMC4004315

[47] Xing Y, et al. Lysosome targeted nanoparticle aggregation reverses immunosuppressive tumor microenvironment for cancer immunotherapy. Adv Mater. 2024;36(47):e2412730. 10.1002/adma.202412730. 39358936 10.1002/adma.202412730

[48] Da Silva J, et al. Radiotherapy-activated NBTXR3 nanoparticles promote ferroptosis through induction of lysosomal membrane permeabilization. J Exp Clin Cancer Res. 2024;43(1):11. 10.1186/s13046-023-02938-0. 38173001 10.1186/s13046-023-02938-0 PMC10762921

[49] Wang J, et al. Polyhedral magnetic nanoparticles induce apoptosis in gastric cancer stem cells and suppressing tumor growth through magnetic force generation. J Control Release. 2024;373:370–84. 10.1016/j.jconrel.2024.07.041. 39032573 10.1016/j.jconrel.2024.07.041

[50] Kim DH, et al. Biofunctionalized magnetic-vortex microdiscs for targeted cancer-cell destruction. Nat Mater. 2010;9(2):165–71. 10.1038/nmat2591. 19946279 10.1038/nmat2591 PMC2810356

[51] Yao J, et al. Nanoplatform-mediated calcium overload for cancer therapy. J Mater Chem B. 2022;10(10):1508–19. 10.1039/d1tb02721b. 35166287 10.1039/d1tb02721b

[52] Hussein-Al-Ali SH, et al. Chlorambucil-iron oxide nanoparticles as a drug delivery system for leukemia cancer cells. Int J Nanomedicine. 2021;16:6205–16. 10.2147/ijn.S312752. 34526768 10.2147/IJN.S312752 PMC8435621

[53] Xu W, et al. Recent advances in chemodynamic nanotherapeutics to overcome multidrug resistance in cancers. Biomed Pharmacother. 2025;184:117901. 10.1016/j.biopha.2025.117901. 39933445 10.1016/j.biopha.2025.117901

[54] Ou R, et al. Advancements in the application of the Fenton reaction in the cancer microenvironment. Pharmaceutics. 2023. 10.3390/pharmaceutics15092337. 37765305 10.3390/pharmaceutics15092337 PMC10536994

[55] Dixon SJ, et al. Ferroptosis: an iron-dependent form of nonapoptotic cell death. Cell. 2012;149(5):1060–72. 10.1016/j.cell.2012.03.042. 22632970 10.1016/j.cell.2012.03.042 PMC3367386

[56] Yang WS, et al. Ferroptosis: death by lipid peroxidation. Trends Cell Biol. 2016;26(3):165–76. 10.1016/j.tcb.2015.10.014. 26653790 10.1016/j.tcb.2015.10.014 PMC4764384

[57] Stockwell BR. Ferroptosis turns 10: emerging mechanisms, physiological functions, and therapeutic applications. Cell. 2022;185(14):2401–21. 10.1016/j.cell.2022.06.003. 35803244 10.1016/j.cell.2022.06.003 PMC9273022

[58] Feng Z, et al. An acid-responsive MOF nanomedicine for augmented anti-tumor immunotherapy via a metal ion interference-mediated pyroptotic pathway. Biomaterials. 2023;302:122333. 10.1016/j.biomaterials.2023.122333. 37738743 10.1016/j.biomaterials.2023.122333

[59] Liu T, et al. *Polyporus umbellatus* polysaccharide iron-based nanocomposite for synergistic M1 polarization of TAMs and combinational anti-breast cancer therapy. Int J Biol Macromol. 2023;251:126323. 10.1016/j.ijbiomac.2023.126323. 37586629 10.1016/j.ijbiomac.2023.126323

[60] Dixon SJ, et al. Ferroptosis: a flexible constellation of related biochemical mechanisms. Mol Cell. 2023;83(7):1030–42. 10.1016/j.molcel.2023.03.005. 36977413 10.1016/j.molcel.2023.03.005 PMC10081971

[61] Dixon SJ, et al. The cell biology of ferroptosis. Nat Rev Mol Cell Biol. 2024;25(6):424–42. 10.1038/s41580-024-00703-5. 38366038 10.1038/s41580-024-00703-5 PMC12187608

[62] Cañeque T, et al. Activation of lysosomal iron triggers ferroptosis in cancer. Nature. 2025;642(8067):492–500. 10.1038/s41586-025-08974-4. 40335696 10.1038/s41586-025-08974-4 PMC12158755

[63] Mao C, et al. DHODH-mediated ferroptosis defence is a targetable vulnerability in cancer. Nature. 2021;593(7860):586–90. 10.1038/s41586-021-03539-7. 33981038 10.1038/s41586-021-03539-7 PMC8895686

[64] Tang D, et al. Ferroptosis in immunostimulation and immunosuppression. Immunol Rev. 2024;321(1):199–210. 10.1111/imr.13235. 37424139 10.1111/imr.13235

[65] Wiernicki B, et al. Cancer cells dying from ferroptosis impede dendritic cell-mediated anti-tumor immunity. Nat Commun. 2022;13(1):3676. 10.1038/s41467-022-31218-2. 35760796 10.1038/s41467-022-31218-2 PMC9237053

[66] Bristow RG, et al. Hypoxia and metabolism. Hypoxia, DNA repair and genetic instability. Nat Rev Cancer. 2008;8(3):180–92. 10.1038/nrc2344. 18273037 10.1038/nrc2344

[67] Ye Y, et al. Characterization of hypoxia-associated molecular features to aid hypoxia-targeted therapy. Nat Metab. 2019;1(4):431–44. 10.1038/s42255-019-0045-8. 31984309 10.1038/s42255-019-0045-8 PMC6980239

[68] Chen Z, et al. Hypoxic microenvironment in cancer: molecular mechanisms and therapeutic interventions. Signal Transduct Target Ther. 2023;8(1):70. 10.1038/s41392-023-01332-8. 36797231 10.1038/s41392-023-01332-8 PMC9935926

[69] Raza A, et al. Pericytes and vessel maturation during tumor angiogenesis and metastasis. Am J Hematol. 2010;85(8):593–8. 10.1002/ajh.21745. 20540157 10.1002/ajh.21745

[70] Estephan H, et al. Hypoxia promotes tumor immune evasion by suppressing MHC-I expression and antigen presentation. EMBO J. 2025;44(3):903–22. 10.1038/s44318-024-00319-7. 39753950 10.1038/s44318-024-00319-7 PMC11790895

[71] Corzo CA, et al. HIF-1α regulates function and differentiation of myeloid-derived suppressor cells in the tumor microenvironment. J Exp Med. 2010;207(11):2439–53. 10.1084/jem.20100587. 20876310 10.1084/jem.20100587 PMC2964584

[72] Chen Y, et al. Iron-based MOF with catalase-like activity improves the synergistic therapeutic effect of PDT/ferroptosis/starvation therapy by reversing the tumor hypoxic microenvironment. J Nanobiotechnol. 2024;22(1):705. 10.1186/s12951-024-02921-7. 10.1186/s12951-024-02921-7 PMC11562077 39543666

[73] Yen TY, et al. Catalase-functionalized iron oxide nanoparticles reverse hypoxia-induced chemotherapeutic resistance. Adv Healthc Mater. 2019;8(20):e1900826. 10.1002/adhm.201900826. 31557421 10.1002/adhm.201900826 PMC6919328

[74] Sen Gupta A. Bio-inspired nanomedicine strategies for artificial blood components. WIREs Nanomed Nanobiotechnol. 2017. 10.1002/wnan.1464. 10.1002/wnan.1464 PMC5599317 28296287

[75] Wang H, et al. Nanoparticles-mediated reoxygenation strategy relieves tumor hypoxia for enhanced cancer therapy. J Control Release. 2020;319:25–45. 10.1016/j.jconrel.2019.12.028. 31862359 10.1016/j.jconrel.2019.12.028

[76] Chen MH, et al. Multifunctional nanoparticles as radiosensitizers to overcome hypoxia-associated resistance in cancer radiotherapy. Nanomaterials (Basel). 2024. 10.3390/nano15010037. 39791794 10.3390/nano15010037 PMC11723374

[77] Lewis CE, et al. Distinct role of macrophages in different tumor microenvironments. Cancer Res. 2006;66(2):605–12. 10.1158/0008-5472.Can-05-4005. 16423985 10.1158/0008-5472.CAN-05-4005

[78] Condeelis J, et al. Macrophages: obligate partners for tumor cell migration, invasion, and metastasis. Cell. 2006;124(2):263–6. 10.1016/j.cell.2006.01.007. 16439202 10.1016/j.cell.2006.01.007

[79] DeNardo DG, et al. Macrophages as regulators of tumour immunity and immunotherapy. Nat Rev Immunol. 2019;19(6):369–82. 10.1038/s41577-019-0127-6. 30718830 10.1038/s41577-019-0127-6 PMC7339861

[80] Xia Y, et al. Engineering macrophages for cancer immunotherapy and drug delivery. Adv Mater. 2020;32(40):e2002054. 10.1002/adma.202002054. 32856350 10.1002/adma.202002054

[81] Horvat NK, et al. Superparamagnetic iron oxide nanoparticles reprogram the tumor microenvironment and reduce lung cancer regrowth after crizotinib treatment. ACS Nano. 2024;18(17):11025–41. 10.1021/acsnano.3c08335. 38626916 10.1021/acsnano.3c08335 PMC11064219

[82] Zanganeh S, et al. Iron oxide nanoparticles inhibit tumour growth by inducing pro-inflammatory macrophage polarization in tumour tissues. Nat Nanotechnol. 2016;11(11):986–94. 10.1038/nnano.2016.168. 27668795 10.1038/nnano.2016.168 PMC5198777

[83] Wang C, et al. Manganese increases the sensitivity of the cGAS-STING pathway for double-stranded DNA and is required for the host defense against DNA viruses. Immunity. 2018;48(4):675-687.e677. 10.1016/j.immuni.2018.03.017. 29653696 10.1016/j.immuni.2018.03.017

[84] Sun X, et al. Amplifying STING activation by cyclic dinucleotide-manganese particles for local and systemic cancer metalloimmunotherapy. Nat Nanotechnol. 2021;16(11):1260–70. 10.1038/s41565-021-00962-9. 34594005 10.1038/s41565-021-00962-9 PMC8595610

[85] Luo G, et al. Multifunctional calcium-manganese nanomodulator provides antitumor treatment and improved immunotherapy via reprogramming of the tumor microenvironment. ACS Nano. 2023;17(16):15449–65. 10.1021/acsnano.3c01215. 37530575 10.1021/acsnano.3c01215 PMC10448754

[86] Wei X, et al. A multifunctional exosome with dual homeostasis disruption augments cGAS-STING-mediated tumor immunotherapy by boosting ferroptosis. Nano Lett. 2024;24(45):14263–72. 10.1021/acs.nanolett.4c03862. 39475013 10.1021/acs.nanolett.4c03862

[87] Li CX, et al. Artificially reprogrammed macrophages as tumor-tropic immunosuppression-resistant biologics to realize therapeutics production and immune activation. Adv Mater. 2019;31(15):e1807211. 10.1002/adma.201807211. 30803083 10.1002/adma.201807211

[88] Liao ZX, et al. Synergistic effect of repolarization of M2 to M1 macrophages induced by iron oxide nanoparticles combined with lactate oxidase. Int J Mol Sci. 2021. 10.3390/ijms222413346. 34948143 10.3390/ijms222413346 PMC8705044

[89] Gammella E, et al. Macrophages: central regulators of iron balance. Metallomics. 2014;6(8):1336–45. 10.1039/c4mt00104d. 24905850 10.1039/c4mt00104d

[90] Recalcati S, et al. Macrophages and iron: a special relationship. Biomedicines. 2021. 10.3390/biomedicines9111585. 34829813 10.3390/biomedicines9111585 PMC8615895

[91] Portilla Y, et al. Interaction of iron oxide nanoparticles with macrophages is influenced distinctly by “Self” and “Non-Self” biological identities. ACS Appl Mater Interfaces. 2023;15(30):35906–26. 10.1021/acsami.3c05555. 37478159 10.1021/acsami.3c05555 PMC10401511

[92] Rojas JM, et al. Superparamagnetic iron oxide nanoparticle uptake alters M2 macrophage phenotype, iron metabolism, migration and invasion. Nanomedicine. 2016;12(4):1127–38. 10.1016/j.nano.2015.11.020. 26733263 10.1016/j.nano.2015.11.020

[93] Laoubi L, et al. Dendritic cells: the central partner for cancer immunity. Oncoimmunology. 2025;14(1):2571773. 10.1080/2162402x.2025.2571773. 41236866 10.1080/2162402X.2025.2571773 PMC12622325

[94] Clayton G, et al. Dendritic cell immunotherapy advances for solid tumors: vaccination and modulation. Cell Rep Med. 2025;6(11):102412. 10.1016/j.xcrm.2025.102412. 41086813 10.1016/j.xcrm.2025.102412 PMC12711664

[95] Chiang M-R, et al. Reprogramming dysfunctional dendritic cells by a versatile catalytic dual oxide antigen-captured nanosponge for remotely enhancing lung metastasis immunotherapy. ACS Nano. 2025;19(2):2117–35. 10.1021/acsnano.4c09525. 39739571 10.1021/acsnano.4c09525 PMC11760334

[96] Huynh TMH, et al. Programmed antigen capture-harnessed dendritic cells by margination-hitchhiking lung delivery. J Control Release. 2023;358:718–28. 10.1016/j.jconrel.2023.05.028. 37230295 10.1016/j.jconrel.2023.05.028

[97] Jiang Q, et al. Platelet membrane-camouflaged magnetic nanoparticles for ferroptosis-enhanced cancer immunotherapy. Small. 2020;16(22):e2001704. 10.1002/smll.202001704. 32338436 10.1002/smll.202001704

[98] Zhang Y, et al. Multifunctional nanoparticle potentiates the in situ vaccination effect of radiation therapy and enhances response to immune checkpoint blockade. Nat Commun. 2022;13(1):4948. 10.1038/s41467-022-32645-x. 35999216 10.1038/s41467-022-32645-x PMC9399096

[99] Alloatti A, et al. Dendritic cell maturation and cross-presentation: timing matters! Immunol Rev. 2016;272(1):97–108. 10.1111/imr.12432. 27319345 10.1111/imr.12432 PMC6680313

[100] Song M, et al. Dendritic cell activation by iron oxide nanoparticles depends on the extracellular environment. Nanoscale Adv. 2024;7(1):209–18. 10.1039/d4na00561a. 39569333 10.1039/d4na00561a PMC11575603

[101] Korangath P, et al. Iron oxide nanoparticles inhibit tumor progression and suppress lung metastases in mouse models of breast cancer. ACS Nano. 2024;18(15):10509–26. 10.1021/acsnano.3c12064. 38564478 10.1021/acsnano.3c12064 PMC11025112

[102] Liang H, et al. Renal clearable ultrasmall single-crystal Fe nanoparticles for highly selective and effective ferroptosis therapy and immunotherapy. J Am Chem Soc. 2021;143(38):15812–23. 10.1021/jacs.1c07471. 34473493 10.1021/jacs.1c07471

[103] Ma X, et al. CD36-mediated ferroptosis dampens intratumoral CD8(+) T cell effector function and impairs their antitumor ability. Cell Metab. 2021;33(5):1001-1012.e1005. 10.1016/j.cmet.2021.02.015. 33691090 10.1016/j.cmet.2021.02.015 PMC8102368

[104] Del Sol-Fernández S, et al. Magnetogenetics: remote activation of cellular functions triggered by magnetic switches. Nanoscale. 2022;14(6):2091–118. 10.1039/d1nr06303k. 35103278 10.1039/d1nr06303k PMC8830762

[105] Pan J, et al. Mild magnetic hyperthermia-activated innate immunity for liver cancer therapy. J Am Chem Soc. 2021;143(21):8116–28. 10.1021/jacs.1c02537. 33928777 10.1021/jacs.1c02537

[106] Kobayashi T, et al. Antitumor immunity by magnetic nanoparticle-mediated hyperthermia. Nanomedicine. 2014;9(11):1715–26. 10.2217/nnm.14.106. 25321171 10.2217/nnm.14.106

[107] Evans SS, et al. Fever and the thermal regulation of immunity: the immune system feels the heat. Nat Rev Immunol. 2015;15(6):335–49. 10.1038/nri3843. 25976513 10.1038/nri3843 PMC4786079

[108] Knippertz I, et al. Mild hyperthermia enhances human monocyte-derived dendritic cell functions and offers potential for applications in vaccination strategies. Int J Hyperthermia. 2011;27(6):591–603. 10.3109/02656736.2011.589234. 21846195 10.3109/02656736.2011.589234

[109] Li Z, et al. Hyperthermia targeting the tumor microenvironment facilitates immune checkpoint inhibitors. Front Immunol. 2020;11:595207. 10.3389/fimmu.2020.595207. 33240283 10.3389/fimmu.2020.595207 PMC7680736

[110] Decraene B, et al. Immunogenic cell death and its therapeutic or prognostic potential in high-grade glioma. Genes Immun. 2022;23(1):1–11. 10.1038/s41435-021-00161-5. 35046546 10.1038/s41435-021-00161-5 PMC8866117

[111] van Rhoon GC, et al. CEM43°C thermal dose thresholds: a potential guide for magnetic resonance radiofrequency exposure levels? Eur Radiol. 2013;23(8):2215–27. 10.1007/s00330-013-2825-y. 23553588 10.1007/s00330-013-2825-y PMC3799975

[112] Behrouzkia Z, et al. Hyperthermia: how can it be used? Oman Med J. 2016;31(2):89–97. 10.5001/omj.2016.19. 27168918 10.5001/omj.2016.19 PMC4861392

[113] Enomoto A, et al. Hyperthermia-mediated cell death via deregulation of extracellular signal-regulated kinase and c-Jun NH2-terminal kinase signaling. Front Cell Death. 2024;3:1465506. 10.3389/fceld.2024.1465506.

[114] Van Dieren L, et al. Combined radiotherapy and hyperthermia: a systematic review of immunological synergies for amplifying radiation-induced abscopal effects. Cancers (Basel). 2024;16(21):3656. 10.3390/cancers16213656. 39518094 10.3390/cancers16213656 PMC11545184

[115] Israel LL, et al. Magnetic iron oxide nanoparticles for imaging, targeting and treatment of primary and metastatic tumors of the brain. J Control Release. 2020;320:45–62. 10.1016/j.jconrel.2020.01.009. 31923537 10.1016/j.jconrel.2020.01.009 PMC7641100

[116] Avasthi A, et al. Magnetic nanoparticles as MRI contrast agents. Top Curr Chem. 2020;378(3):40. 10.1007/s41061-020-00302-w. 10.1007/s41061-020-00302-w PMC8203530 32382832

[117] Chalise D, et al. Highly sensitive and high-throughput magnetic resonance thermometry of fluids using superparamagnetic nanoparticles. Phys Rev Appl. 2023;19(1):014055. 10.1103/PhysRevApplied.19.014055.

[118] Gao R, et al. An intracellular magnetic micromotor drives spatiotemporal mechano-immunotherapy. Adv Mater. 2025;37(47):e04404. 10.1002/adma.202504404. 40944369 10.1002/adma.202504404

[119] Guillemin PC, et al. Using the tissue impulse response function to streamline fractionated MRgFUS-induced hyperthermia. Cancers (Basel). 2025;17(3):515. 10.3390/cancers17030515. 39941882 10.3390/cancers17030515 PMC11817472

[120] Roudi R, et al. Novel clinically translatable iron oxide nanoparticle for monitoring anti-CD47 cancer immunotherapy. Invest Radiol. 2024;59(5):391–403. 10.1097/rli.0000000000001030. 37812494 10.1097/RLI.0000000000001030 PMC10997482

[121] Huang Y, et al. The metabolic fate of iron-based magnetic nanomaterials and their impact on macrophage function. Magnetic Medicine. 2025;1(1):100002. 10.1016/j.magmed.2025.100002.

[122] Arami H, et al. In vivo delivery, pharmacokinetics, biodistribution and toxicity of iron oxide nanoparticles. Chem Soc Rev. 2015;44(23):8576–607. 10.1039/c5cs00541h. 26390044 10.1039/c5cs00541h PMC4648695

[123] Volatron J, et al. Ferritin protein regulates the degradation of iron oxide nanoparticles. Small. 2017. 10.1002/smll.201602030. 28060465 10.1002/smll.201602030

[124] Gao J, et al. Time-course effect of ultrasmall superparamagnetic iron oxide nanoparticles on intracellular iron metabolism and ferroptosis activation. Nanotoxicology. 2021;15(3):366–79. 10.1080/17435390.2021.1872112. 33455495 10.1080/17435390.2021.1872112

[125] Koleini N, et al. Ironing out mechanisms of iron homeostasis and disorders of iron deficiency. J Clin Invest. 2021. 10.1172/jci148671. 34060484 10.1172/JCI148671 PMC8159681

[126] Kluknavsky M, et al. A single infusion of polyethylene glycol-coated superparamagnetic magnetite nanoparticles alters differently the expressions of genes involved in iron metabolism in the liver and heart of rats. Pharmaceutics. 2023;15(5):1475. 10.3390/pharmaceutics15051475. 37242717 10.3390/pharmaceutics15051475 PMC10220547

[127] Singh N, et al. Potential toxicity of superparamagnetic iron oxide nanoparticles (SPION). Nano Rev. 2010;1. 10.3402/nano.v1i0.5358. 10.3402/nano.v1i0.5358 PMC3215220 22110864

[128] Matusiak K, et al. The elemental changes occurring in the rat liver after exposure to PEG-coated iron oxide nanoparticles: total reflection x-ray fluorescence (TXRF) spectroscopy study. Nanotoxicology. 2017;11(9–10):1225–36. 10.1080/17435390.2017.1408151. 29183205 10.1080/17435390.2017.1408151

[129] Unterweger H, et al. Comparative in vitro and in vivo evaluation of different iron oxide-based contrast agents to promote clinical translation in compliance with patient safety. Int J Nanomedicine. 2023;18:2071–86. 10.2147/ijn.S402320. 37113796 10.2147/IJN.S402320 PMC10128873

[130] Lartigue L, et al. Biodegradation of iron oxide nanocubes: high-resolution in situ monitoring. ACS Nano. 2013;7(5):3939–52. 10.1021/nn305719y. 23634880 10.1021/nn305719y

[131] Portilla Y, et al. Different coatings on magnetic nanoparticles dictate their degradation kinetics in vivo for 15 months after intravenous administration in mice. J Nanobiotechnol. 2022;20(1):543. 10.1186/s12951-022-01747-5. 10.1186/s12951-022-01747-5 PMC9795732 36578018

[132] Wilhelm S, et al. Analysis of nanoparticle delivery to tumours. Nat Rev Mater. 2016;1(5):16014. 10.1038/natrevmats.2016.14.

[133] Cheng YH, et al. Meta-analysis of nanoparticle delivery to tumors using a physiologically based pharmacokinetic modeling and simulation approach. ACS Nano. 2020;14(3):3075–95. 10.1021/acsnano.9b08142. 32078303 10.1021/acsnano.9b08142 PMC7098057

[134] Mitchell MJ, et al. Engineering precision nanoparticles for drug delivery. Nat Rev Drug Discov. 2021;20(2):101–24. 10.1038/s41573-020-0090-8. 33277608 10.1038/s41573-020-0090-8 PMC7717100

[135] Dai Q, et al. Quantifying the ligand-coated nanoparticle delivery to cancer cells in solid tumors. ACS Nano. 2018;12(8):8423–35. 10.1021/acsnano.8b03900. 30016073 10.1021/acsnano.8b03900

[136] Li Y, et al. Specific surface-modified iron oxide nanoparticles trigger complement-dependent innate and adaptive antileukaemia immunity. Nat Commun. 2024;15(1):10400. 10.1038/s41467-024-54810-0. 39613769 10.1038/s41467-024-54810-0 PMC11607078

[137] Liu H, et al. Cell membrane-coated nanoparticles: a novel multifunctional biomimetic drug delivery system. Drug Deliv Transl Res. 2023;13(3):716–37. 10.1007/s13346-022-01252-0. 36417162 10.1007/s13346-022-01252-0 PMC9684886

[138] Healy S, et al. Spatial analysis of nanoparticle distribution in human breast xenografts reveals nanoparticles targeted to cancer cells localized with tumor-associated stromal cells. Nanotheranostics. 2023;7(4):393–411. 10.7150/ntno.84255. 37426881 10.7150/ntno.84255 PMC10327423

[139] Joyce P, et al. A translational framework to DELIVER nanomedicines to the clinic. Nat Nanotechnol. 2024;19(11):1597–611. 10.1038/s41565-024-01754-7. 39242807 10.1038/s41565-024-01754-7

[140] Valcourt DM, et al. Best practices for preclinical in vivo testing of cancer nanomedicines. Advanced Healthcare Materials. 2020;9(12):e2000110. 10.1002/adhm.202000110. 32367687 10.1002/adhm.202000110 PMC7473451

[141] Liu Y, et al. Patient-derived xenograft models in cancer therapy: technologies and applications. Signal Transduct Target Ther. 2023;8(1):160. 10.1038/s41392-023-01419-2. 37045827 10.1038/s41392-023-01419-2 PMC10097874

[142] Bosenberg M, et al. Mouse models for immuno-oncology. Trends Cancer. 2023;9(7):578–90. 10.1016/j.trecan.2023.03.009. 37087398 10.1016/j.trecan.2023.03.009

[143] Jiang W, et al. Considerations for designing preclinical cancer immune nanomedicine studies. Nat Nanotechnol. 2021;16(1):6–15. 10.1038/s41565-020-00817-9. 33349682 10.1038/s41565-020-00817-9 PMC8103921

[144] Bruni D, et al. The immune contexture and immunoscore in cancer prognosis and therapeutic efficacy. Nat Rev Cancer. 2020;20(11):662–80. 10.1038/s41568-020-0285-7. 32753728 10.1038/s41568-020-0285-7

[145] Martin JD, et al. Improving cancer immunotherapy using nanomedicines: progress, opportunities and challenges. Nat Rev Clin Oncol. 2020;17(4):251–66. 10.1038/s41571-019-0308-z. 32034288 10.1038/s41571-019-0308-z PMC8272676

[146] Liu YT, et al. Turning cold tumors into hot tumors by improving T-cell infiltration. Theranostics. 2021;11(11):5365–86. 10.7150/thno.58390. 33859752 10.7150/thno.58390 PMC8039952

[147] Coulton A, et al. Using a pan-cancer atlas to investigate tumour associated macrophages as regulators of immunotherapy response. Nat Commun. 2024;15(1):5665. 10.1038/s41467-024-49885-8. 38969631 10.1038/s41467-024-49885-8 PMC11226649

[148] Nam J, et al. Cancer nanomedicine for combination cancer immunotherapy. Nat Rev Mater. 2019;4(6):398–414. 10.1038/s41578-019-0108-1.

[149] Kwon M, et al. The right timing, right combination, right sequence, and right delivery for cancer immunotherapy. J Control Release. 2021;331:321–34. 10.1016/j.jconrel.2021.01.009. 33434599 10.1016/j.jconrel.2021.01.009

[150] Stribbling SM, et al. Orthotopic and metastatic tumour models in preclinical cancer research. Pharmacol Ther. 2024;257:108631. 10.1016/j.pharmthera.2024.108631. 38467308 10.1016/j.pharmthera.2024.108631 PMC11781865

[151] Boone CE, et al. Combining nanomedicine and immune checkpoint therapy for cancer immunotherapy. WIREs Nanomed Nanobiotechnol. 2022;14(1):e1739. 10.1002/wnan.1739. 10.1002/wnan.1739 PMC8906799 34296535

[152] Peske JD, et al. Control of CD8 T-cell infiltration into tumors by vasculature and microenvironment. Adv Cancer Res. 2015;128:263–307. 10.1016/bs.acr.2015.05.001. 26216636 10.1016/bs.acr.2015.05.001 PMC4638417

[153] Giraldo NA, et al. The clinical role of the TME in solid cancer. Br J Cancer. 2019;120(1):45–53. 10.1038/s41416-018-0327-z. 30413828 10.1038/s41416-018-0327-z PMC6325164

[154] Zhou Z, et al. Evaluation of ferroptosis as a biomarker to predict treatment outcomes of cancer immunotherapy. Cancer Res Commun. 2025;5(8):1288–97. 10.1158/2767-9764.Crc-25-0268. 40693608 10.1158/2767-9764.CRC-25-0268 PMC12326525

[155] U.S. Food and Drug Administration. FERABRIGHT (ferumoxytol injection) [prescribing information]. U.S. Food and Drug Administration; 2025.

[156] Li Y, et al. Clinical translation and landscape of superparamagnetic iron oxide nanoparticles. Adv Drug Deliv Rev. 2026;229:115756. 10.1016/j.addr.2025.115756. 41360153 10.1016/j.addr.2025.115756

[157] Rodriguez B, et al. Magnetic hyperthermia therapy for high-grade glioma: a state-of-the-art review. Pharmaceuticals (Basel). 2024. 10.3390/ph17030300. 38543086 10.3390/ph17030300 PMC10976112

[158] Pallares RM, et al. Clinical cancer nanomedicines. J Control Release. 2025;385:113991. 10.1016/j.jconrel.2025.113991. 40582644 10.1016/j.jconrel.2025.113991

[159] Gabizon AA, et al. Thirty years from FDA approval of pegylated liposomal doxorubicin (Doxil/Caelyx): an updated analysis and future perspective. BMJ Oncol. 2025;4(1):e000573. 10.1136/bmjonc-2024-000573. 39885941 10.1136/bmjonc-2024-000573 PMC11751825

[160] AMAG Pharmaceuticals, Inc. Feraheme (ferumoxytol injection) [prescribing information]. Waltham, MA: AMAG Pharmaceuticals, Inc.; 2022.

[161] Kok HP, et al. Heating technology for malignant tumors: a review. Int J Hyperthermia. 2020;37(1):711–41. 10.1080/02656736.2020.1779357. 32579419 10.1080/02656736.2020.1779357 PMC7781160

